# Association of COVID-19 With Major Arterial and Venous Thrombotic Diseases: A Population-Wide Cohort Study of 48 Million Adults in England and Wales

**DOI:** 10.1161/CIRCULATIONAHA.122.060785

**Published:** 2022-09-20

**Authors:** Rochelle Knight, Venexia Walker, Samantha Ip, Jennifer A. Cooper, Thomas Bolton, Spencer Keene, Rachel Denholm, Ashley Akbari, Hoda Abbasizanjani, Fatemeh Torabi, Efosa Omigie, Sam Hollings, Teri-Louise North, Renin Toms, Xiyun Jiang, Emanuele Di Angelantonio, Spiros Denaxas, Johan H. Thygesen, Christopher Tomlinson, Ben Bray, Craig J. Smith, Mark Barber, Kamlesh Khunti, George Davey Smith, Nishi Chaturvedi, Cathie Sudlow, William N. Whiteley, Angela M. Wood, Jonathan A.C. Sterne

**Affiliations:** Department of Population Health Sciences, Bristol Medical School, University of Bristol, UK (R.K., V.W., J.A.C., R.D., T.-L.N., R.T., G.D.S., J.A.C.S.).; NIHR Bristol Biomedical Research Centre, UK (R.K., J.A.C., R.D., J.A.C.S.).; NIHR Applied Research Collaboration West, Bristol, UK (R.K.).; MRC Integrative Epidemiology Unit, Bristol, UK (R.K., V.W., G.D.S.).; British Heart Foundation Cardiovascular Epidemiology Unit (S.I., T.B., S.K., X.J., E.D.A., A.M.W.), University of Cambridge, UK.; Centre for Cancer Genetic Epidemiology (S.I.), University of Cambridge, UK.; Department of Public Health and Primary Care, NIHR Blood and Transplant Research Unit in Donor Health and Genomics (T.B., S.K., E.D.A., A.M.W.), University of Cambridge, UK.; British Heart Foundation Centre of Research Excellence (E.D.A., A.M.W.), University of Cambridge, UK.; British Heart Foundation Data Science Centre (T.B., C.S.), London.; Health Data Research UK (S.D.), London.; Health Data Research UK South-West, Bristol (R.D., J.A.C.S.).; Population Data Science, Swansea University Medical School, Swansea University, Wales, UK (A.A., H.A., F.T.).; National Health Service Digital, Leeds, UK (E.O., S.H.).; School of Health Sciences, Cardiff Metropolitan University, UK (R.T.).; Wellcome Genome Campus, Health Data Research UK Cambridge (E.D.A., A.M.W.).; Institute of Health Informatics (S.D., J.H.T., C.T.), University College London, UK.; UK Research and Innovation Centre for Doctoral Training in AI-Enabled Healthcare Systems (C.T.), University College London, UK.; University College London Hospitals Biomedical Research Centre (C.T., S.D.), University College London, UK.; MRC Unit for Lifelong Health and Ageing at UCL, Institute of Cardiovascular Science (N.C.), University College London, UK.; BHF Accelerator, London, UK (S.D.).; School of Population Health and Environmental Sciences, King’s College London, UK (B.B.).; Geoffrey Jefferson Brain Research Centre, Manchester Centre for Clinical Neurosciences, Northern Care Alliance National Health Service Foundation Trust, Salford Royal Hospital, UK (C.J.S.).; Division of Cardiovascular Sciences, Manchester Academic Health Science Centre, University of Manchester, UK (C.J.S.).; Glasgow Caledonian University, UK (M.B.).; Diabetes Research Centre, University of Leicester, UK (K.K.).; Centre for Clinical Brain Sciences, University of Edinburgh, UK (W.N.W.).; Nuffield Department of Population Health, University of Oxford, UK (W.N.W.).; NIHR Cambridge Biomedical Research Centre, UK (A.M.W.).; Cambridge Centre for AI in Medicine, UK (A.M.W.).

**Keywords:** COVID-19, electronic health records, myocardial infarction, pulmonary embolism, stroke, thrombosis, venous thrombosis

## Abstract

Infection with severe acute respiratory syndrome coronavirus 2 (SARS-CoV-2) induces a prothrombotic state, but long-term effects of COVID-19 on incidence of vascular diseases are unclear.

**Methods:**

We studied vascular diseases after COVID-19 diagnosis in population-wide anonymized linked English and Welsh electronic health records from January 1 to December 7, 2020. We estimated adjusted hazard ratios comparing the incidence of arterial thromboses and venous thromboembolic events (VTEs) after diagnosis of COVID-19 with the incidence in people without a COVID-19 diagnosis. We conducted subgroup analyses by COVID-19 severity, demographic characteristics, and previous history.

**Results:**

Among 48 million adults, 125 985 were hospitalized and 1 319 789 were not hospitalized within 28 days of COVID-19 diagnosis. In England, there were 260 279 first arterial thromboses and 59 421 first VTEs during 41.6 million person-years of follow-up. Adjusted hazard ratios for first arterial thrombosis after COVID-19 diagnosis compared with no COVID-19 diagnosis declined from 21.7 (95% CI, 21.0–22.4) in week 1 after COVID-19 diagnosis to 1.34 (95% CI, 1.21–1.48) during weeks 27 to 49. Adjusted hazard ratios for first VTE after COVID-19 diagnosis declined from 33.2 (95% CI, 31.3–35.2) in week 1 to 1.80 (95% CI, 1.50–2.17) during weeks 27 to 49. Adjusted hazard ratios were higher, for longer after diagnosis, after hospitalized versus nonhospitalized COVID-19, among Black or Asian versus White people, and among people without versus with a previous event. The estimated whole-population increases in risk of arterial thromboses and VTEs 49 weeks after COVID-19 diagnosis were 0.5% and 0.25%, respectively, corresponding to 7200 and 3500 additional events, respectively, after 1.4 million COVID-19 diagnoses.

**Conclusions:**

High relative incidence of vascular events soon after COVID-19 diagnosis declines more rapidly for arterial thromboses than VTEs. However, incidence remains elevated up to 49 weeks after COVID-19 diagnosis. These results support policies to prevent severe COVID-19 by means of COVID-19 vaccines, early review after discharge, risk factor control, and use of secondary preventive agents in high-risk patients.

**Clinical Perspective**

**What Is New?**

- In a cohort study of 48 million adults in England and Wales, COVID-19 was associated with substantial excess incidence of both arterial thromboses and venous thromboembolism, which declined with time from COVID-19 diagnosis.
- Excess incidence was higher, for a longer time, after hospitalized than nonhospitalized COVID-19.
- There were an estimated 10 500 excess arterial thromboses and venous thromboembolic events after 1.4 million COVID-19 diagnoses.

**What Are the Clinical Implications?**

- Strategies to prevent vascular events after COVID-19 are particularly important after severe COVID-19 leading to hospitalization and should include an early review in primary care and risk factor management.
- After severe COVID-19, individuals at high risk of vascular events should be prescribed preventive therapies and counseled about the importance of adherence to them.
- New simple treatment strategies to reduce infection-associated venous thromboembolism and arterial thromboses are needed.

Infection with severe acute respiratory syndrome coronavirus 2 (SARS-CoV-2), which causes COVID-19, induces a prothrombotic and proinflammatory state that may increase the risk of serious thrombotic disorders.^1^ Most previous studies suggest immediate marked increases in arterial (largely myocardial infarction [MI] and stroke) and venous thromboembolic events (VTEs),^2–8^ although the numbers may be exaggerated because of universal testing for COVID-19 in all hospital admissions (including for thrombosis), surveillance for venous thrombosis in COVID-19 cohorts, or underuse of thromboprophylaxis. However, few studies have quantified long-term vascular risks after diagnosis of COVID-19 or explored how these risks differ by key characteristics such as age, sex, race, or preexisting comorbidities.

Anonymized population-scale linked primary and secondary care electronic health records for the population of England and Wales were analyzed to compare the incidence of major arterial and venous thromboses in people with and without a diagnosis of COVID-19, accounting for multiple potential confounding factors. These comparisons were also made in men and women, different age groups, and different racial groups. We estimated the relative incidence of thrombotic events in people who were and were not hospitalized with COVID-19 compared with people without a diagnosis of COVID-19.

## Methods

Procedures for accessing the data analyzed in this article are described by CVD-COVID-UK/COVID-IMPACT and SAIL (Secure Anonymised Information Linkage) Databank. The analysis was performed according to a prespecified protocol and analysis plan with phenotyping and analysis code (available at github.com/BHFDSC/CCU002_01). R. Knight had full access to all the data in the study and takes responsibility for its integrity and that of the data analysis.

### Data Sharing

Data used in this study are available in the National Health Service (NHS) Digital Trusted Research Environment (TRE) for England (https://digital.nhs.uk/coronavirus/coronavirus-data-services-updates/trusted-research-environment-service-for-england); because restrictions apply, they are not publicly available. The CVD-COVID-UK/COVID-IMPACT program led by the British Heart Foundation Data Science Centre (https://www.hdruk.ac.uk/helping-with-health-data/bhf-data-science-centre) received approval to access data in the NHS Digital TRE for England from the Independent Group Advising on the Release of Data (https://digital.nhs.uk/about-nhs-digital/corporate-information-and-documents/independent-group-advising-on-the-release-of-data) through an application made in the Data Access Request Service Online system (reference DARS-NIC-381078-Y9C5K; https://digital.nhs.uk/services/data-access-request-service-dars/dars-products-and-services). The CVD-COVID-UK/COVID-IMPACT Approvals and Oversight Board (https://www.hdruk.ac.uk/projects/cvd-covid-uk-project) subsequently granted approval to this project to access the data within the NHS Digital TRE for England and the SAIL Databank. The deidentified data used in this study were made available to accredited researchers only. Those wishing to gain access to the data should contact bhfdsc@hdruk.ac.uk.

Data used in this study are available in the SAIL Databank at Swansea University, United Kingdom; because restrictions apply, they are not publicly available. All proposals to use SAIL data are subject to review by an independent Information Governance Review Panel. Before any data can be accessed, approval must be given by the Information Governance Review Panel. The Information Governance Review Panel gives careful consideration to each project to ensure proper and appropriate use of SAIL data. When access has been granted, it is gained through a privacy protecting data safe haven and remote access system referred to as the SAIL Gateway. SAIL has established an application process to be followed by anyone who would like to access data through SAIL at https://www.saildatabank.com/application-process.

### Population

Pseudonymized data on adults alive and registered with a primary care general practice in England or Wales on January 1, 2020, were accessed and analyzed through the British Heart Foundation Data Science Centre CVD-COVID-UK/COVID-IMPACT consortium within the NHS Digital secure, privacy-protecting TRE Service for England and the SAIL Databank for Wales.^9,10^ The TRE for England includes primary care data (General Practice Extraction Service data for Pandemic Planning and Research) from 98% of general practices linked at individual level to secondary care data including all NHS hospital admissions, critical care, emergency department, and outpatient visits (Hospital Episode Statistics and Secondary Uses Service data from 1997 onwards), COVID-19 laboratory testing data, national community drug dispensing data (NHS Business Services Authority Dispensed Medicines from 2018), and death registrations. The SAIL Databank includes data from hospital admissions, mortality registers, primary care, COVID-19 test results, community dispensing, and critical care, enabled through the C19_Cohort20 platform.^11^

### COVID-19 Diagnosis

COVID-19 diagnosis was defined as a record of a positive COVID-19 polymerase chain reaction or antigen test or a confirmed COVID-19 diagnosis in primary care or secondary care hospital admission records and we derived the earliest date on which COVID-19 was recorded (Table S1). A confirmed COVID-19 diagnosis did not require documentation of a positive test for SARS-CoV-2 because widespread testing was not available in the United Kingdom until October 2020. Hospitalization for COVID-19 was defined as a hospital admission record with confirmed COVID-19 diagnosis in the primary position of electronic hospital records within 28 days of first COVID-19 diagnosis and COVID-19 without hospitalization as a COVID-19 diagnosis that was not followed by hopitalization within 28 days. Hospitalization for COVID-19 was classified further as with and without critical care within 28 days of COVID-19 diagnosis. COVID-19 critical care was defined on the basis of receipt of noninvasive ventilation, invasive mechanical ventilation or extracorporeal membrane oxygenation, or admission to an intensive care unit.^12^ Events after hospitalized COVID-19 were classified into those happening during the hospital admission and those happening after discharge. We used the latest available discharge date for the admission. If no discharge date was available, the latest episode end date relating to the admission was used. Rates of events during and after hospital admission were quantified as number of events per 1000 person-years.

### Outcomes

Outcomes were defined using primary care, hospital admission, and national death registry data (Tables S2 and S3). Specialist clinician-verified SNOMED-CT (Systematized Nomenclature of Medicine–Clinical Terms), Read code, and ICD-10 (International Classification of Diseases, 10th Revision) rule-based phenotyping algorithms were used to define fatal or nonfatal arterial thromboses (MI, ischemic stroke [ischemic or unclassified stroke, spinal stroke, or retinal infarction], or other nonstroke non-MI arterial thromboembolism); VTEs (pulmonary embolism [PE], lower limb deep venous thrombosis [DVT], other DVT, portal vein thrombosis, or intracranial venous thrombosis); and other vascular events (transient ischemic attack, hemorrhagic stroke [intracerebral or subarachnoid hemorrhage], heart failure, or angina). An outcome event was defined as fatal if it was followed by death from any cause within 28 days. All remaining outcome events were defined as nonfatal. Patients with thromboembolic disease were treated according to national guidelines.^13–15^

### Potential Confounding Variables

Primary and secondary care records up to January 1, 2020, were used to define race, deprivation, smoking status, and region. A large number of potentially confounding variables were defined on the basis of previous disease diagnoses, comorbidities, and medications (Table S4).

### Statistical Analyses

We estimated hazard ratios (HRs) comparing the incidence of arterial thromboses, VTEs, and other vascular events after a diagnosis of COVID-19 with the incidence of these events in people without a diagnosis of COVID-19 (the reference group, which was combined from people with no record of COVID-19 during follow-up and follow-up time before COVID-19 in those who developed COVID-19 during follow-up). We estimated HRs in separate time periods after diagnosis of COVID-19 (0 to 6 days and 1 to 2, 3 to 4, 5 to 8, 9 to 12, 13 to 26, and 27 to 49 weeks since diagnosis). Analyses used Cox regression models with calendar time scale (starting on January 1, 2020) to account for rapid changes in the incidence of COVID-19, fitted separately by age group (categorized as <40, 40 to 59, 60 to 79 and ≥80 years on January 1, 2020) and by population (England and Wales). Censoring was at the earliest of the date of the outcome, death, or December 7, 2020 (the day before the UK COVID-19 vaccine rollout started). For computational efficiency, analyses included all people with the outcome of interest or with a record of COVID-19 infection and a 10% randomly sampled subset of other people. Analyses incorporated inverse probability weights with robust standard errors to account for this sampling. Overall HRs were combined across age groups using inverse-variance weighted meta-analyses.

We estimated age-, sex-, and region-adjusted and maximally adjusted HRs: the latter controlled for all the potential confounders listed in Table S4. Where necessary in subgroup analyses, potential confounders with ≤2 disease events at any level were excluded. In subgroup analyses for which there were no outcome events in one or more time periods after COVID-19 diagnosis, the time periods were collapsed into categories 1 to 4 and >5 weeks since COVID-19 diagnosis.

Separate analyses were conducted to estimate HRs for hospitalized and nonhospitalized COVID-19 compared with no COVID-19. For the combined arterial thrombosis and VTE outcomes, additional subgroup analyses were conducted by sex, race, and history of arterial thrombosis and VTE, respectively. Because of the smaller population size, analyses of Welsh data excluded the <40 years age group, were restricted to all COVID-19 diagnoses and the combined arterial thrombosis and VTE outcomes, and were conducted separately only by sex. These results were combined across the English and Welsh populations using inverse-variance weighted meta-analyses.

We estimated HRs using separate models for age group and, in the relevant analyses, for hospitalized and nonhospitalized COVID-19. Overall results were then derived by combining HRs across age groups using inverse-variance meta-analysis. The same set of covariates was adjusted for in each age group before results were combined. In some analyses, limited numbers of outcome events after COVID-19 meant that the younger age groups had to be combined to fit maximally adjusted models. For some models examining outcomes after hospitalized COVID-19, all age groups had to be combined because small numbers of outcome events after hospitalized COVID-19 made it impossible to identify a set of covariates that could be adjusted for across all groups, or some regions had to be merged.

The average daily incidence of major arterial thromboses and VTEs before or in the absence of COVID-19 was calculated across the whole follow-up period, separately in subgroups defined by age and sex. These were multiplied by the maximally adjusted age- and sex-specific HR for that day to derive the incidence on each day after COVID-19. A life table approach was used to calculate age- and sex-specific cumulative risks over time with and without COVID-19 and the latter was subtracted from the former to derive the absolute excess risks over time after COVID-19 compared with no COVID-19 diagnosis. Overall absolute excess risk was estimated from a weighted sum of the age- and sex-specific excess risks, weighted by the proportions of people in age and sex strata within the COVID-19–infected population in England during the follow-up period.

### Study Oversight

Approval for the CVD-COVID-UK/COVID-IMPACT research program to access, within secure trusted research environments, unconsented, whole-population, deidentified data from electronic health records collected as part of patients’ routine health care was obtained from the Newcastle & North Tyneside 2 Research Ethics Committee (20/NE/0161), the NHS Digital Data Access Request Service (DARS-NIC 381078-Y9C5K), and the SAIL independent Information Governance Review Panel (project number 0911) and for this project from the British Heart Foundation Data Science Centre CVD-COVID-UK/COVID-IMPACT Approvals and Oversight Board. Analyses used SQL, Python, and RStudio (Professional) Version 1.3.1093.1 driven by R Version 4.0.3 (2020-10-10).

## Results

Among 44 964 486 people in the population of England, 118 879 (264/100 000) were hospitalized with COVID-19 (22 992 of these patients received critical care) and 1 248 180 (2776/100 000) were not hospitalized within 28 days of their COVID-19 diagnosis (Table 1). Of 51 949 deaths from COVID-19 after a COVID-19 diagnosis, 37 908 were in those hospitalized with COVID-19: a further 6764 COVID-19 deaths were recorded in people with no COVID-19 diagnosis. Among 2 615 854 people in the Wales population, 7106 (272/100 000) and 71 606 (2737/100 000), respectively, were hospitalized and not hospitalized after COVID-19 diagnosis (Table S5).

The risk of nonhospitalized COVID-19 was higher in women than men (3110 versus 2428/100 000), but the risk of hospitalized COVID-19 was higher in men than women (304 versus 226/100 000; Table 1). As expected, the risk of hospitalized COVID-19 increased markedly with increasing age, from 28.3/100 000 at age 18 to 29 years to 1944/100 000 at age 90+ years. By contrast, the risk of nonhospitalized COVID-19 was higher (3816 and 4749/100 000) in these youngest and oldest age groups and lowest (1450/100 000) in those 70 to 79 years of age. The risks of both hospitalized and nonhospitalized COVID-19 increased with increasing index of multiple deprivation.

**Table 1.**
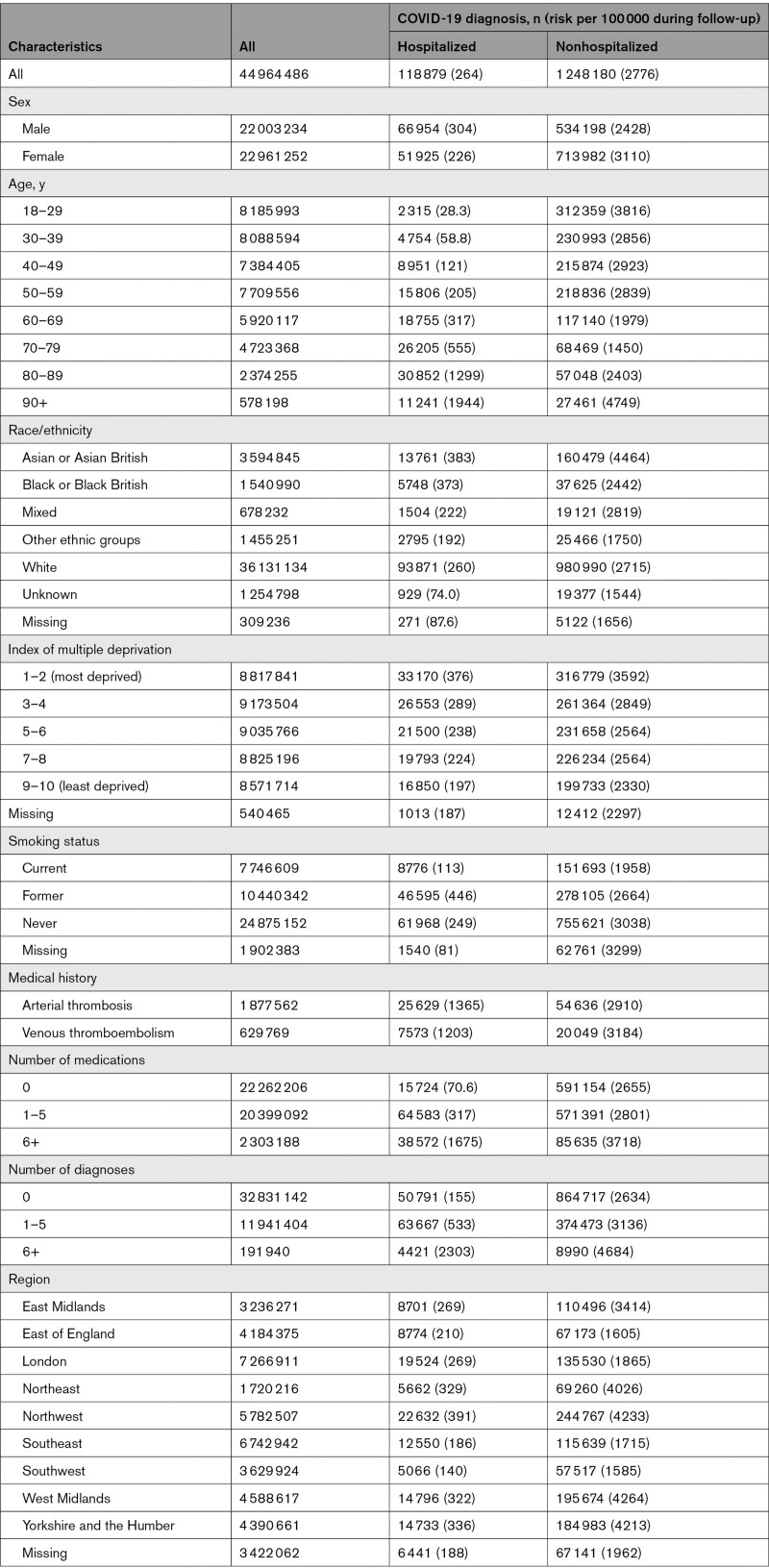
People Analyzed in the English Trusted Research Environment and Patients Who Were and Were Not Hospitalized Within 28 Days of COVID-19 Diagnosis

Numbers of arterial thromboses, VTEs, and other vascular events before COVID-19 and after hospitalized and nonhospitalized COVID-19 in the England population are shown in Table 2. Of 260 279 arterial thromboses, 2241 were in the 118 879 patients hospitalized with COVID-19 (317 and 1924 with and without critical care, respectively), and 5180 were in the 1 248 180 patients who were not hospitalized with COVID-19. Corresponding figures for VTEs were 59 421 total events, 800 (114 and 686 with and without critical care) among hospitalized patients and 1808 among nonhospitalized patients. A total of 1726 (5.3%) of 32 622 fatal arterial thromboses occurred after a COVID-19 diagnosis, compared with 5695 (2.5%) of 227 657 nonfatal arterial thromboses. A total of 269 (4.7%) of 5771 fatal VTEs occurred after a COVID-19 diagnosis, compared with 2339 (4.4%) of 53 650 nonfatal VTEs.

**Table 2.**
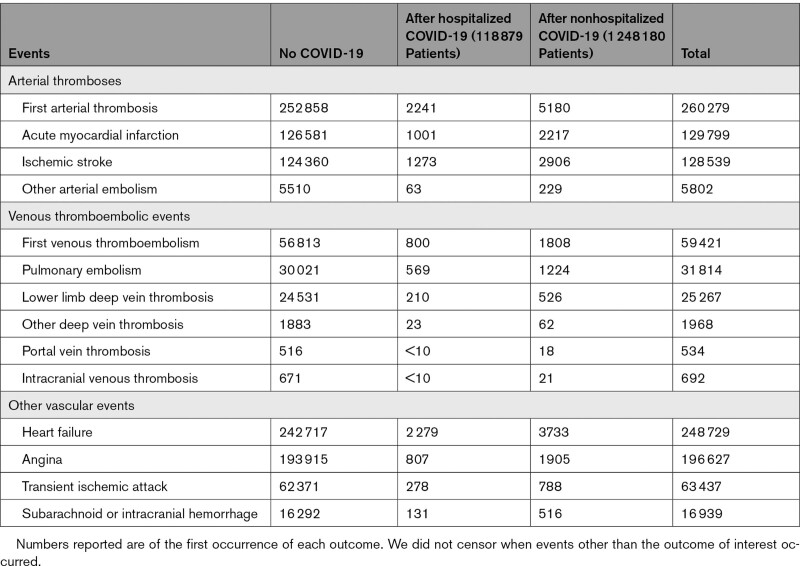
Arterial Thrombotic, Venous Thromboembolic, and Other Vascular Events in the English Trusted Research Environment Before and After COVID-19 Diagnosis

The median (interquartile range) length of hospital admission was 11 days (6–19). Of 2241 arterial thromboses among patients hospitalized with COVID-19, 1169 were during the hospital admission (272/1000 person-years) and 1072 were after discharge (39.8/1000 person-years). Of 800 venous thromboses, 198 (44.4/1000 person-years) were during the hospital admission and 602 (21.8/1000 person-years) were after discharge.

Most arterial thromboses were either acute MI (129 799) or ischemic stroke (128 539) and most VTEs were either PE (31 814) or lower limb DVT (25 267). The proportion of strokes attributable to hemorrhage was as expected (9.3% after hospitalized COVID-19 and 15.1% after nonhospitalized COVID-19). The total person-years of follow-up in the England population were 41 595 372 before COVID-19, 32 471 after hospitalized COVID-19, and 245 817 after nonhospitalized COVID-19. Corresponding figures in the Wales population were 2 383 967, 1709, and 12 966.

**Figure 1.**
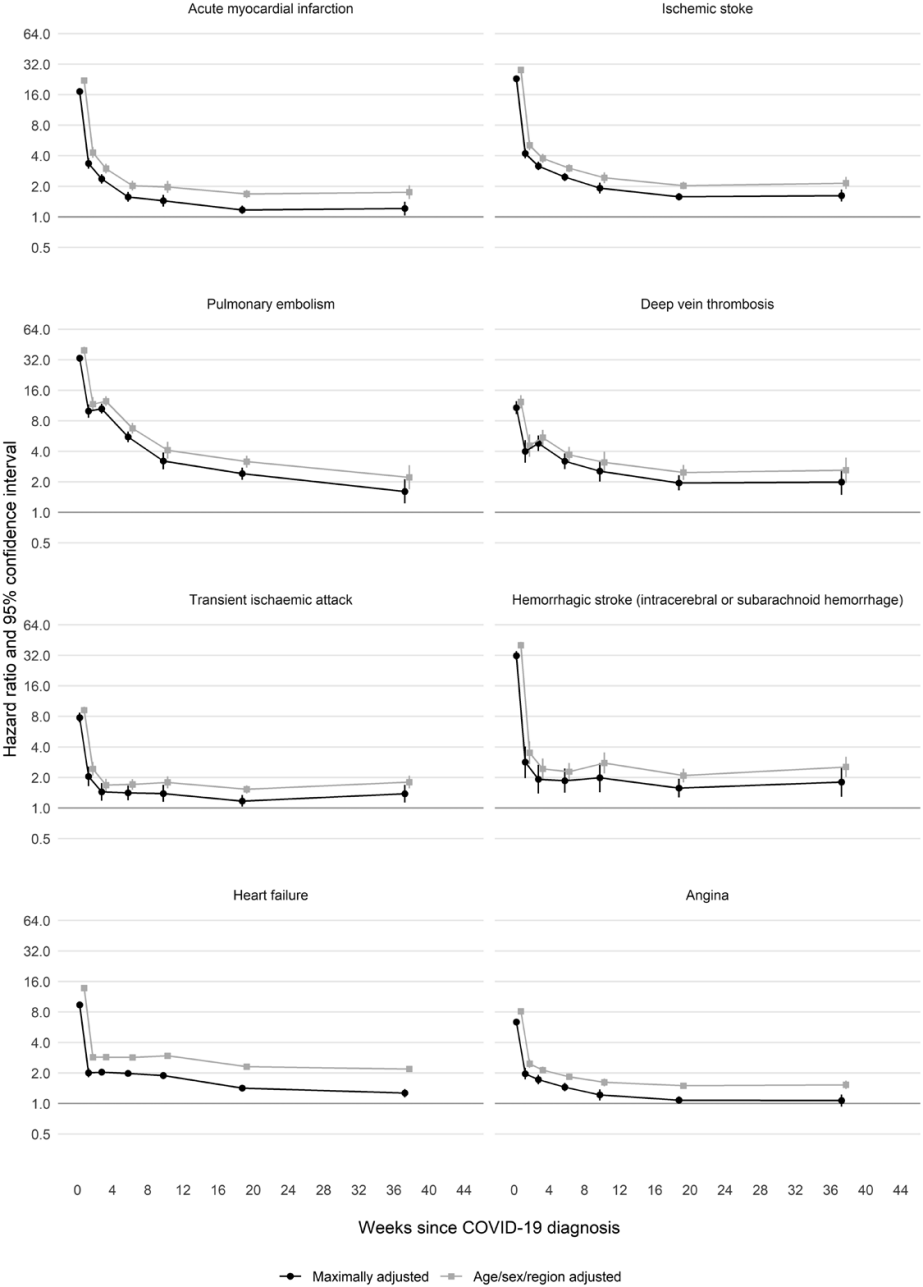
**Age-, sex-, and region-adjusted and maximally adjusted hazard ratios (log scale) for different arterial thrombotic and venous thromboembolic and other vascular events by time since diagnosis of COVID-19.** All results are maximally adjusted unless otherwise stated.

Across outcomes and all time periods after COVID-19, maximally adjusted HRs (aHRs) were attenuated compared with age-/sex-/region-adjusted HRs (Figure 1 and Table 3). aHRs for acute MI declined rapidly from 17.2 (95% CI,16.3–18.1) in week 1 to 1.21 (1.03–1.41) in weeks 27 to 49. They were higher, for longer after diagnosis, after hospitalized compared with nonhospitalized COVID-19: aHRs during weeks 27 to 49 were 1.39 (1.12–1.72) and 1.03 (0.83–1.28), respectively. aHRs for ischemic stroke were higher than for MI: they declined from 28.1 (26.8–29.4) in week 1 to 1.62 (1.42–1.86) in weeks 27 to 49, at which time they were 1.62 (1.33–1.98) and 1.33 (1.10–1.59) after hospitalized and nonhospitalized COVID-19, respectively (Figure S1).

**Table 3.**
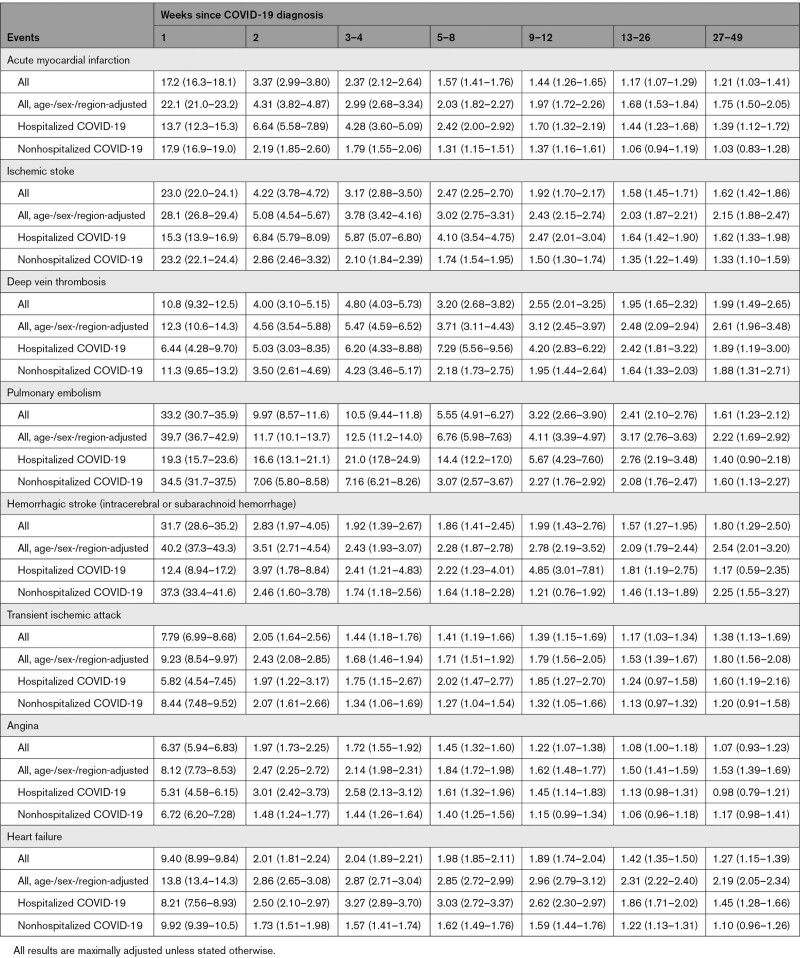
Hazard Ratios (95% CI) Compared With No COVID-19 for Different Arterial Thromboses, Venous Thromboembolism Events, and Other Vascular Events According to Time Since COVID-19 Diagnosis

Rates of DVT and PE were elevated for longer after diagnosis of COVID-19 than those for arterial thromboses: aHRs compared with no COVID-19 were 4.80 (95% CI, 4.03–5.73) and 10.5 (9.44–11.8), respectively, 3 to 4 weeks after diagnosis, declining to 1.62 (1.42–1.86) and 1.99 (1.49–2.65) in weeks 27 to 49, by which time aHRs were similar for hospitalized and nonhospitalized COVID-19. Overall aHRs for hemorrhagic stroke declined to <2 by 3 to 4 weeks after diagnosis, but aHRs after hospitalized COVID-19 peaked again (aHR, 4.85 [3.01–7.81]) 9 to 12 weeks after diagnosis. aHRs for angina and heart failure declined rapidly and were <1.5 by 13 to 26 weeks after diagnosis.

**Figure 2.**
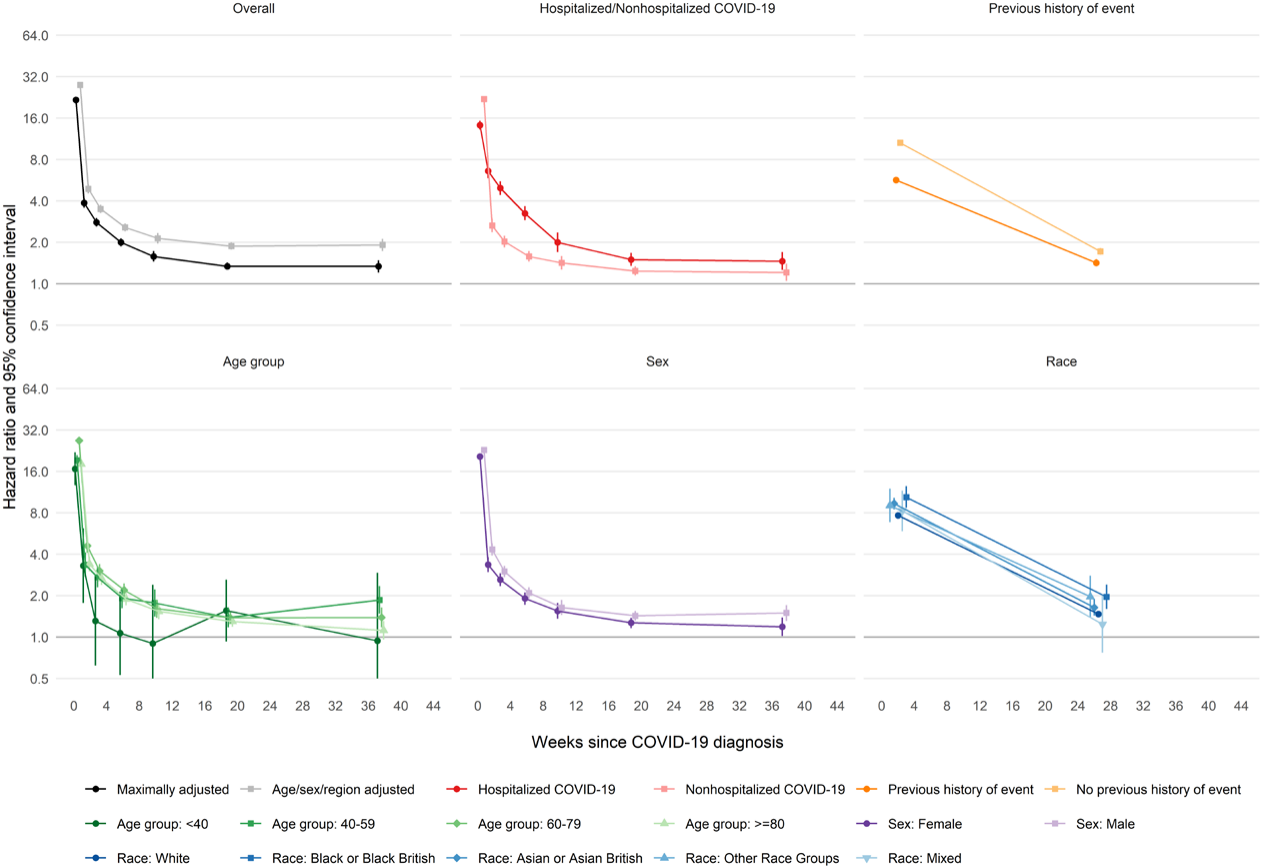
Hazard ratios (log scale) for first arterial event after COVID-19 by time since diagnosis, overall and stratified by whether hospitalized with COVID-19, previous history of an arterial event, age, sex, and race. All results are maximally adjusted unless otherwise stated.

For the first (of any) arterial thrombosis, aHRs compared with no COVID-19 declined rapidly from 21.7 (95% CI, 21.0–22.4) to 3.87 (3.58–4.19) between the first and second weeks after COVID-19 to 2.80 (2.61–3.01) during weeks 3 to 4 and then more gradually to 1.34 (1.21–1.48) during weeks 27 to 49 (Figure 2 and Table 4). aHRs were higher after hospitalized than nonhospitalized COVID-19 from week 2 (6.60 [5.85–7.44] versus 2.65 [2.37–2.96]) onwards, declining to 1.46 (1.26–1.70) versus 1.21 (1.05–1.40) by weeks 27 to 49. From 4 weeks after diagnosis onwards, aHRs for arterial thromboses were substantially higher (compared with no COVID-19) in hospitalized patients who had received critical care than in those who had not (Figure S2 and Table S6). aHRs were higher for fatal arterial thromboses after COVID-19 (compared with no COVID-19) than for nonfatal arterial thromboses (Figure S3 and Table S6).

**Table 4.**
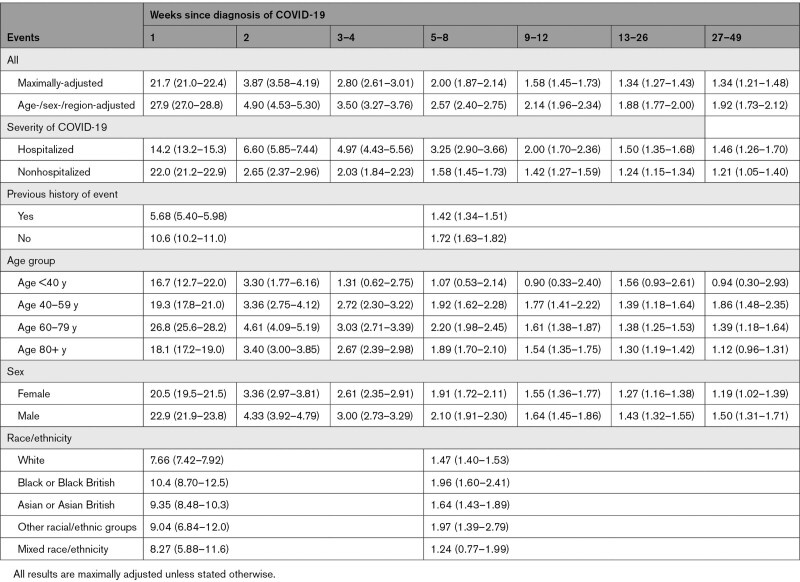
Hazard Ratios (95% CI) Compared With No COVID-19 for First Arterial Thrombosis According to Time Since COVID-19 Diagnosis

During weeks 1 to 4, aHRs were greater in those with no previous history of arterial thrombosis (12.1 [11.5–12.8]) compared with those with a history of arterial thrombosis (6.21 [5.30–7.27]), an effect that attenuated with duration of follow-up. There were no consistent differences between age groups. aHRs were marginally greater in males than females. aHRs were greater in Black or Black British people (10.4 [95% CI, 8.70–12.5] and 1.96 [1.60–2.41] during weeks 1 to 4 and 5 to 49, respectively) and Asian or Asian British people (9.35 [8.48–10.30] and 1.64 [1.43–1.89], respectively) than in White people (7.66 [7.42–7.92] and 1.47 [1.40–1.53], respectively).

**Figure 3.**
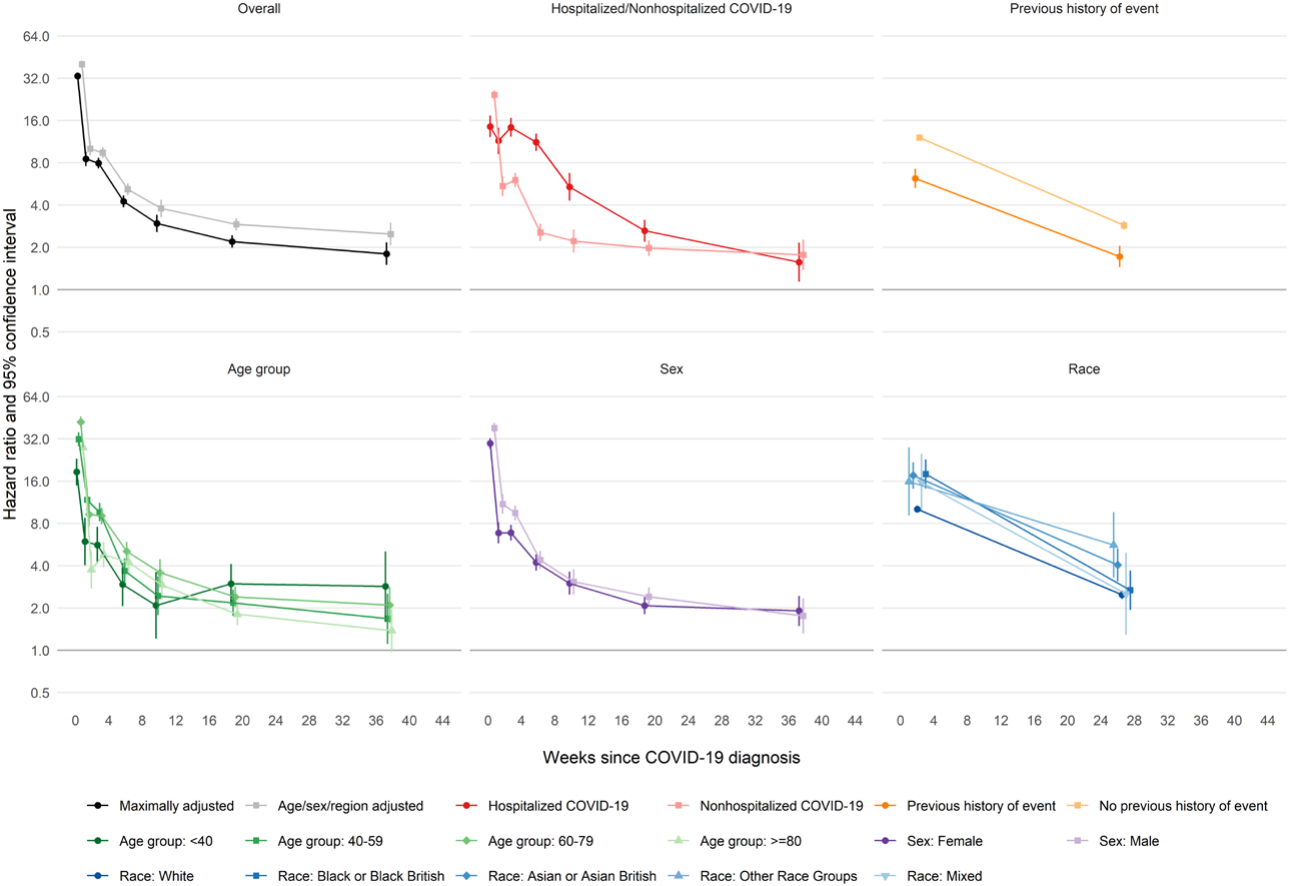
Hazard ratios for first venous thromboembolism after COVID-19 by time since diagnosis, overall and stratified by whether hospitalized with COVID-19, previous history of a venous thromboembolism, age, sex, and race. All results are maximally adjusted unless otherwise stated.

For the first VTE, aHRs after COVID-19 compared with no COVID-19 declined more gradually than those for arterial thromboses, from 33.2 (95% CI, 31.3–35.2) and 8.52 (7.59–9.58) in the first and second weeks to 7.95 (7.28–8.68) and 4.26 (3.86–4.69) during weeks 3 to 4 and 5 to 8, then more gradually to 2.20 (1.99–2.44) and 1.80 (1.50–2.17) during weeks 13 to 26 and 27 to 49, respectively (Figure 3 and Table 5). aHRs for VTEs were substantial for the first 8 weeks after hospitalized COVID-19 (11.2 [9.72–12.9] during weeks 5 to 8, then 5.40 [4.31–6.77], 2.63 [2.19–3.14], and 1.57 [1.14–2.16] during weeks 9 to 12, 13 to 26, and 27 to 49, respectively). After nonhospitalized COVID-19, aHRs were 2.56 (2.22–2.95), 2.22 (1.84–2.68), 1.98 (1.74–2.25), and 1.77 (1.38–2.27) during weeks 5 to 8, 9 to 12, 13 to 26, and 27 to 49, respectively. From 4 weeks after diagnosis onwards, aHRs for VTEs were initially higher (compared with no COVID-19) in hospitalized patients who had received critical care than in those who had not, but there was little evidence that they differed from 13 weeks after diagnosis (Figure S2). In contrast with the findings for arterial thromboses, aHRs for fatal VTEs after COVID-19 (compared with no COVID-19) were lower than for nonfatal VTEs (Figure S3 and Table S6).

**Table 5.**
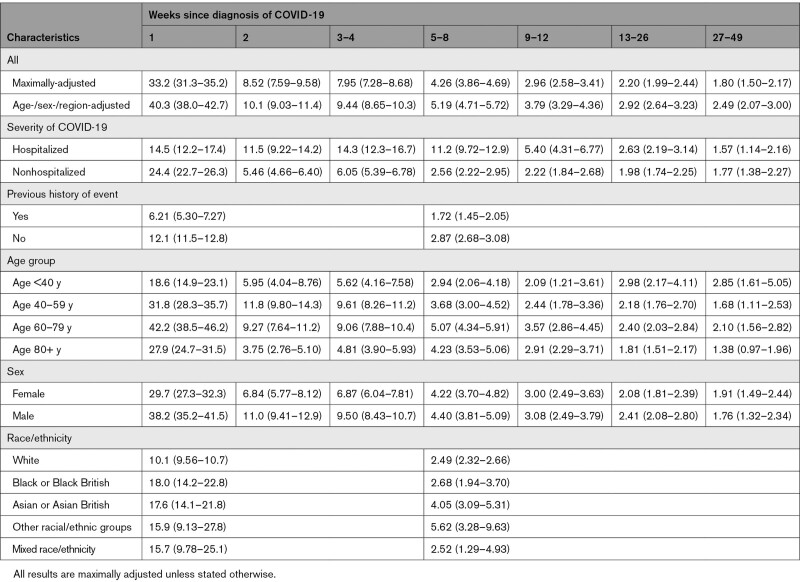
Hazard Ratios (95% CI) Compared With No COVID-19 for First Venous Thromboembolism According to Time Since COVID-19 Diagnosis

aHRs were greater in those without than with a history of VTE, but did not differ markedly between age groups. aHRs in males were greater than those in females during weeks 1 to 4 after COVID-19 diagnosis. aHRs were higher in people of Black or Black British race (18.0 [14.3–22.8] and 2.68 [1.94–3.70] during weeks 1 to 4 and 5 to 49, respectively) and people of Asian or Asian British race (17.6 [14.2–21.8] and 4.05 [3.09–5.31], respectively) than those of White race (10.1 [9.56–10.7] and 2.49 [2.32–2.66], respectively).

**Figure 4.**
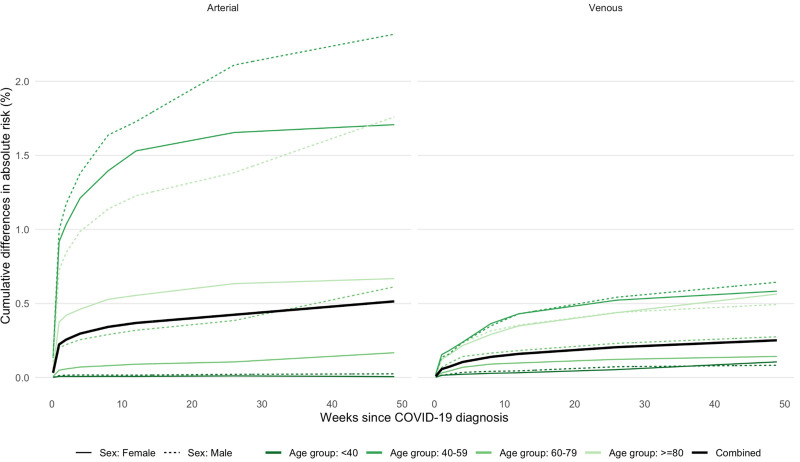
Estimated absolute increase in risk of arterial thrombosis and venous thromboembolism over time since diagnosis of COVID-19 compared with no COVID-19 diagnosis.

Absolute excess risks were generally greater in men and in older people (Figure 4). Combining all arterial thromboses, the excess risk 49 weeks after diagnosis of COVID-19 ranged from 2.3% and 1.7%, respectively, in men and women ≥80 years of age to 0.03% and 0.01%, respectively, in men and women <40 years of age (Figure 4). Combining all VTEs, the excess risk at 49 weeks ranged from 0.6% in men and women ≥80 years of age to 0.1% in men and women <40 years of age. Excluding events in the first 28 days approximately halved these absolute excess risks (Figure S4). Figure S5 shows that 49 weeks after diagnosis, the excess risks of both arterial thromboses and VTEs were higher for hospitalized COVID-19 (0.7% and 0.4%, respectively) than for nonhospitalized COVID-19 (0.4% and 0.2%, respectively). Across the whole population, the estimated absolute increases in the risk of arterial thromboses and VTEs were 0.5% and 0.25%, respectively. This corresponds to 7200 and 3500 additional arterial thromboses and VTEs, respectively, after 1.4 million COVID-19 diagnoses.

## Discussion

In this cohort of 48 million adults, a markedly higher incidence of arterial thromboses in the first weeks after COVID-19 diagnosis, relative to no COVID-19 diagnosis, declined rapidly with time after diagnosis. The excess incidence of VTEs in the first weeks after COVID-19 diagnosis declined less rapidly than for arterial thromboses and was twofold higher for up to 49 weeks after COVID-19 diagnosis. For both arterial thromboses and VTEs, relative incidence was higher, and remained elevated for longer, after hospitalized than nonhospitalized COVID-19. Associations between COVID-19 and thrombotic events did not vary markedly by age or sex, but were greater in people of Black or Asian race than those of White race, and in people without than with a history of vascular events. We estimate that by December 2020, COVID-19 led to >10 500 additional arterial thromboses and VTEs in England and Wales.

Like other studies of vascular disease risk after COVID-19 infection, ^4,7,8,16^ this study found that incidence of arterial thromboses and VTEs was markedly elevated in the first 1 to 2 weeks after COVID-19 diagnosis and declined with time from diagnosis. Two self-controlled case series studies found that excluding cases of arterial thromboses or VTEs recorded on the first day of COVID-19 diagnosis attenuated the early relative incidence associated with COVID-19.^4,7^ This may have been attributable to ascertainment of COVID-19 at the time of hospitalization for a vascular event or to limited resolution of date coding of COVID-19 and vascular events in the same hospital admission.

Incidence of arterial thromboses and VTEs is also elevated after non–COVID-19 infections. In general, the relative increases in these events are greatest soon after infection and fall within a month toward baseline, although elevated incidence of VTEs may persist for longer. The mechanism for this may relate to persistence of a postinfection inflammatory response that predominantly affects the venous rather than arterial circulation, although whether this is predominantly driven by endothelial, leukocyte, or other components of inflammation is not clear.^17^ Relative increases after non–COVID-19 infections have been shown to be similar to this study’s estimates 2 weeks after COVID-19 diagnosis.^18–21^ Hospital admissions attributable to MI^22^ or stroke^23^ fell during the height of the COVID-19 pandemic in England and Wales, which suggests that increases in hospitalizations for arterial thromboses or VTEs after COVID-19 were small compared with the substantial reductions in diagnoses and health care use at that time.

The large number of COVID-19 infections in England and Wales during 2020 and 2021 is likely to have caused a substantial additional burden of arterial thromboses and VTEs. Strategies to prevent vascular events after COVID-19 will therefore be important at a population level. These will include early review in primary care, risk factor management, and ensuring adherence and preventative therapies in high-risk individuals. However, during the pandemic, there have been substantial reductions in primary care contacts for a number of conditions, including cardiometabolic diseases. These reductions have limited the ability of primary health care professionals to provide routine health checks for people with chronic conditions.^24,25^ The pandemic also led to reductions in prescriptions of antihypertensives and lipid-lowering medication and many patients will have missed reviews of vascular risk modification after COVID-19 diagnosis.^26^

Randomized trials of short courses of antithrombotic interventions with a low risk of harm might be the next step to reduce longer-term risks after hospital discharge. Emerging evidence for this approach is promising,^27^ although its longer-term benefits are uncertain. Observational studies suggest a protective effect of statins and blood pressure lowering against post–COVID-19 vascular events.^28,29^ The risks of arterial thromboses could be mitigated by review of known vascular risk factors soon after a COVID-19 diagnosis.

The excess incidence of thromboembolic events was higher in Black or Asian people than in White people. Black or Asian people have had higher rates of COVID-19 mortality, which appears to be related to geography, deprivation, occupation, household composition, living arrangements, and preexisting health conditions.^30^ Prepandemic differences in control of vascular risk factors between racial groups may also explain these findings.^31^

Our study has a number of strengths. We included almost all of the adult English and Welsh populations; therefore, the results reflect the total population effect of COVID-19 on the incidence of major vascular events, and are generalizable to other settings with comprehensive health care. Linkage with primary care records and national COVID-19 testing data allowed us to study vascular diseases after both hospitalized and nonhospitalized COVID-19 and adjust for a wide range of potentially confounding factors. We used a widely agreed-on set of codes in electronic health records to identify arterial thromboses and VTEs recorded in the first position in hospital and death records. The protocol was prespecified and all code lists are available.

This study has several limitations. First, the survival analyses allowed for variations in diagnoses with calendar time, so should control for the reductions in hospital attendance during the period of maximum disruption (March and April 2020). However, some vascular events may not have been recorded either because patients died in nursing homes with few diagnostic resources or were so unwell that MI, stroke, PE, or DVT diagnoses would have been difficult to make.

Second, people may have avoided health care facilities after minor vascular events because of fear of COVID-19. If this was more likely in people without COVID-19, then estimated HRs would have been biased upwards.

Third, because the English primary care dataset did not include information on PE and DVT, the incidence of milder nonhospitalized VTEs may have been underestimated.

Fourth, we had limited resolution to determine the date order of COVID-19 diagnosis and arterial thromboses or VTEs for some hospitalized patients. Some patients hospitalized with a vascular event either developed a nosocomial infection or had a COVID-19 diagnosis after routine testing on admission. For some patients, a raised troponin level with COVID-19 may have led to a diagnosis of MI.^31^ Therefore, the very high HRs within 1 week of COVID-19 diagnosis may have been inflated by reverse causality.

Fifth, there was underascertainment of COVID-19 infection before testing for SARS-CoV-2 became widely available for mild or asymptomatic infections. Such underdiagnosis would bias estimated post–COVID-19 HRs toward the null.

Sixth, identification of exposures, covariates, and outcomes relies on the accuracy of data recorded in electronic health records during routine health care and we were unable to validate these against fuller health records.

Seventh, unmeasured confounding may explain some findings, because there is a substantial overlap between risk factors for vascular disease and COVID-19. Risk factors for vascular events (eg, body mass index) are not systematically recorded for all patients and are subject to measurement error. The difference between maximally-adjusted and age-/sex-/region-adjusted HRs was more marked longer after COVID-19 diagnosis: the HRs for major arterial events >13 weeks (HR 1.3) after diagnosis could be attributable to unmeasured confounding. However, the higher HRs for VTEs after 13 weeks are less plausibly explained by unmeasured confounding and are consistent with the risk of VTEs after other infections.^18^

### Conclusions

Substantial increases in the relative incidence of arterial thromboses and VTEs 1 to 2 weeks after diagnosis of COVID-19 decline with time since diagnosis, although doubling of the incidence of VTEs persisted for up to 49 weeks after diagnosis. These results support continued policies to prevent severe COVID-19 with effective COVID-19 vaccines, early review and management of vascular risks in patients with COVID-19, and use of secondary preventive agents in patients at high risk of vascular diseases. New simple treatment strategies to reduce infection-associated VTE and arterial thromboses are needed.

## Article Information

### Acknowledgments

This study makes use of deidentified data held in the National Health Service Digital Trusted Research Environment for England (made available through the British Heart Foundation Data Science Center CVD-COVID-UK/COVID-IMPACT consortium); anonymized data held in the SAIL (Secure Anonymised Information Linkage) Databank; and data provided by patients and collected by the National Health Service as part of their care and support. The authors thank the data providers who make health-relevant data available for research. The authors acknowledge the collaborative partnership that enabled acquisition and access to the deidentified data, which led to this output. The collaboration was led by the Swansea University Health Data Research UK team under the direction of the Welsh Government Technical Advisory Cell and includes the following groups and organizations: the SAIL Databank, Administrative Data Research Wales, Digital Health and Care Wales, Public Health Wales, National Health Service Shared Services Partnership, and the Welsh Ambulance Service Trust. All research was conducted under the permission and approval of the SAIL independent Information Governance Review Panel (project number 0911). Dr Whiteley conceived the study. Drs Whiteley, Wood, Denholm, Cooper, Sudlow, and Sterne drafted the protocol. Drs Sterne, Wood, Walker, Cooper, and Denholm designed the statistical analyses. R. Knight, Drs Walker, Ip, and Bolton, S. Keene, A. Akbari, Dr Abbasizanjani, F. Torabi, E. Omigie, Drs Hollings, Denaxas, and Thygesen, and C. Tomlinson developed code lists and derived datasets. Drs Denaxas and Thygesen and C. Tomlinson created electronic health record phenotyping algorithms for hospitalized and nonhospitalized COVID-19. R. Knight, Drs Walker, Ip, Cooper, and Bolton, S. Keene, Dr Denholm, A. Akbari, Dr Abbasizanjani, F. Torabi, Drs North, Toms, Denaxas, and Thygesen, C. Tomlinson, X. Jiang, and Dr Wood conducted statistical analyses. Drs Sterne, Whiteley, Wood, and Walker, R. Knight, and Dr Ip produced the first draft of the manuscript. Dr Sudlow is Director of the British Heart Foundation Data Science Center and coordinated approvals for and access to data within the National Health Service Digital Trusted Research Environment for England and the SAIL Databank Trusted Research Environment for CVD-COVID-UK/COVID-IMPACT. All authors critically appraised the manuscript for important intellectual content and contributed to the final draft of the manuscript.

### Sources of Funding

This work was funded by the Longitudinal Health and Wellbeing COVID-19 National Core Study, which was established by the UK Chief Scientific Officer in October 2020 and funded by UK Research and Innovation (grant references MC_PC_20030 and MC_PC_20059); by the British Heart Foundation as part of the British Heart Foundation Data Science Center led by Health Data Research UK (British Heart Foundation grant number SP/19/3/34678); by the Data and Connectivity National Core Study led by Health Data Research UK in partnership with the Office for National Statistics and funded by UK Research and Innovation (grant reference MC_PC_20058); by the CONVALESCENCE study of long COVID-19 funded by National Institute for Health and Care Research (NIHR)/UK Research and Innovation; by the Con-COV team funded by the Medical Research Council (grant number MR/V028367/1); by Health Data Research UK, which receives its funding from Health Data Research UK Ltd (HDR-9006) funded by the UK Medical Research Council, Engineering and Physical Sciences Research Council, Economic and Social Research Council, Department of Health and Social Care (England), Chief Scientist Office of the Scottish Government Health and Social Care Directorates, Health and Social Care Research and Development Division (Welsh Government), Public Health Agency (Northern Ireland), British Heart Foundation, and the Wellcome Trust; by core funding from the British Heart Foundation (RG/13/13/30194; RG/18/13/33946), British Heart Foundation Cambridge CRE (RE/13/6/30180), and NIHR Cambridge Biomedical Research Center (BRC-1215-20014); by the ADR Wales program of work, which is aligned to the priority themes as identified in the Welsh Government’s national strategy: Prosperity for All (ADR Wales brings together data science experts at Swansea University Medical School, staff from the Wales Institute of Social and Economic Research, Data and Methods at Cardiff University, and specialist teams within the Welsh Government to develop new evidence that supports Prosperity for All by using the SAIL Databank at Swansea University to link and analyze anonymized data; ADR Wales is part of the Economic and Social Research Council [part of UK Research and Innovation] funded ADR UK [grant ES/S007393/1]); by the Wales COVID-19 Evidence Center, funded by Health and Care Research Wales; and by the BigData@Heart Consortium, funded by the Innovative Medicines Initiative-2 Joint Undertaking under grant agreement 116074. Dr Ip was funded by a British Heart Foundation–Turing Cardiovascular Data Science 419 Award (BCDSA/100005) and is funded by the International Alliance for Cancer Early Detection, a partnership among Cancer Research UK C18081/A31373, Canary Center at Stanford University, the University of Cambridge, OHSU Knight Cancer Institute, University College London, and the University of Manchester. R. Knight and Drs Cooper and Sterne were supported by the NIHR Bristol Biomedical Research Center. R. Knight and Drs Walker and Davey Smith were supported by the Medical Research Council Integrative Epidemiology Unit at the University of Bristol (MC_UU_00011/1). R. Knight was supported by NIHR ARC West. Drs Denholm and Sterne were supported by Health Data Research UK. S. Keene is funded by the NIHR Blood and Transplant Research Unit in Donor Health and Genomics (NIHR BTRU-2014-10024). X. Jiang was funded by the Health Data Research UK–Turing Wellcome PhD Programme in Health Data Science. Dr Wood was supported by the British Heart Foundation–Turing Cardiovascular Data Science Award (BCDSA/100005). Dr Whiteley is supported by the Chief Scientist’s Office (CAF/01/17). Drs Sudlow, Smith, Barber, Wood, and Whiteley are supported by the Stroke Association (SA CV 20/100018). C. Tomlinson is supported by a University College London UK Research and Innovation Center for Doctoral Training in AI-Enabled Healthcare studentship (EP/S021612/1), Medical Research Council Clinical Top-Up, and a studentship from the NIHR Biomedical Research Center at University College London Hospital National Health Service Trust. The views expressed are those of the authors and not necessarily those of the NIHR or the Department of Health and Social Care.

### Disclosures

Dr Whiteley has given expert testimony to UK courts and served on an advisory board for Bayer. Dr Chaturvedi receives funds from AstraZeneca to support membership of Data Safety and Monitoring Committees for clinical trials. The other authors report no conflicts.

### Supplemental Material

Tables S1–S6

Figures S1–S5

## Supplementary Material


