# Supplementary material for: Association of COVID-19 With Major Arterial and Venous Thrombotic Diseases: A Population-Wide Cohort Study of 48 Million Adults in England and Wales
Source: Circulation. 2022 Sep 20;146(12):892–906. doi: 10.1161/CIRCULATIONAHA.122.060785 (PMC9484653; doi:10.1161/CIRCULATIONAHA.122.060785)
Supplement: Supplementary file 1 [file cir-146-892-s001.pdf]

SUPPLEMENTAL MATERIAL

Tables S1-S6

Figures S1-S5

Supplementary Table 1. Derivation of COVID-19 variables in the English NHS Digital TRE and Welsh SAIL Databank TRE environments.

All codelists are available in the GitHub for England: https://github.com/BHFDSC/CCU002_01/tree/main/england/phenotypes

and similarly for Wales where Read codes were used for primary care: https://github.com/BHFDSC/CCU002_01/tree/main/Wales/phenotypes

Variable	Data Sources (NHS England)	Data Sources (SAIL Databank)	Description	Variable name in analysis code
**Exposure**				
COVID-19 diagnosis date	SGSS, GDPPR, deaths registry, HES APC, HES CC, CHESS, SUS	PATD (Pillar 1 & 2), PEDW, WLGP	Minimum date of confirmed COVID-19 event post index date. A record of a positive COVID-19 PCR antigen test or a record of confirmed diagnosis in primary care general practice or secondary care hospital admission records.	exp_confirmed_covid19_date
COVID-19 infection severity	SGSS, GDPPR, deaths registry, HES APC, HES CC, CHESS, SUS	PATD (Pillar 1 & 2), PEDW, WLGP	COVID-19 infection with hospitalisation was defined as a hospital admission record with confirmed COVID-19 diagnosis in the primary position within 28 days of first COVID-19 infection. COVID-19 infection without hospitalisation was defined as a confirmed COVID-19 diagnosis in the primary position within 28 days of first COVID-19 infection without a hospital admission record.	exp_confirmed_covid_phenotype

Acronyms: GDPPR (General Practice Extraction Service (GPES) Data for pandemic planning and research), HES (Hospital Episode Statistics), APC (Admitted Patient Care), , ADDE (Annual District Death Extract), PEDW (Patient Episode Dataset for Wales), WLGP (Welsh Longitudinal GP Dataset - Welsh Primary Care), PATD (COVID-19 Test Results), CHESS (COVID-19 Hospitalisation in England Surveillance System), SGSS (Covid-19 Second Generation Surveillance System).

UOB Open

Supplementary Table 2. Derivation of major outcomes in the English NHS Digital TRE and Welsh SAIL Databank TRE environments.

All codelists are available in the GitHub for England https://github.com/BHFDSC/CCU002_01/tree/main/england/phenotypes and similarly for Wales where Read codes were used for primary care: https://github.com/BHFDSC/CCU002_01/tree/main/Wales/phenotypes

Outcome	Data Sources (NHS Digital)	Data Sources (SAIL Databank)	Description	Variable name in analysis code
**Arterial thromboses**				
Myocardial infarction	GDPPR, HES APC, deaths registry	WLGP, PEDW, ADDE	Earliest recorded date of primary care event or consultation, admission to hospital (coded as primary cause), or death (coded as underlying cause).	out_AMI
Ischaemic stroke	GDPPR, HES APC, deaths registry	WLGP, PEDW, ADDE	Earliest recorded date of primary care event or consultation, admission to hospital (coded as primary cause), or death (coded as underlying cause).	out_stroke_isch
Other arterial thromboembolism	HES APC, deaths registry	PEDW, ADDE	Earliest recorded date of admission to hospital (coded as primary cause of the care episode), or death (coded as underlying cause).	out_other_arterial_embolism
**Venous thromboembolic events**				
Pulmonary embolism	HES APC, deaths registry	PEDW, ADDE	Earliest recorded date of admission to hospital (coded as primary cause of the care episode), or death (coded as underlying cause).	out_PE
Lower limb deep vein thrombosis	HES APC, deaths registry	PEDW, ADDE	Earliest recorded date of admission to hospital (coded as primary cause of the care episode), or death (coded as underlying cause).	out_DVT_DVT, out_DVT_pregnancy
Other deep vein thrombosis	HES APC, deaths registry	PEDW, ADDE	Earliest recorded date of admission to hospital (coded as primary cause of the care episode), or death (coded as underlying cause).	out_other_DVT
Portal vein thrombosis	HES APC, deaths registry	PEDW, ADDE	Earliest recorded date of admission to hospital (coded as primary cause of the care episode), or death (coded as underlying cause).	out_portal_vein_thrombosis
Intracranial venous thrombosis	HES APC, deaths registry	PEDW, ADDE	Earliest recorded date of admission to hospital (coded as primary cause of the care episode), or death (coded as underlying cause).	out_ICVT, out_ICVT_pregnancy
**Other vascular events**				
Intracranial or subarachnoid haemorrhage	HES APC, deaths registry	PEDW, ADDE	Earliest recorded date of admission to hospital (coded as primary cause of the care episode), or death (coded as underlying cause).	out_stroke_SAH_HS
Heart failure	GDPPR, HES APC, deaths registry	WLGP, PEDW, ADDE	Earliest recorded date of primary care event or consultation, admission to hospital (coded as primary cause), or death (coded as underlying cause).	out_HF
Angina	GDPPR, HES APC, deaths registry	WLGP, PEDW, ADDE	Earliest recorded date of primary care event or consultation, admission to hospital (coded as primary cause), or death (coded as underlying cause).	out_angina
Transient ischemic attack	GDPPR, HES APC, deaths registry	WLGP, PEDW, ADDE	Earliest recorded date of primary care event or consultation, admission to hospital (coded as primary cause), or death (coded as underlying cause).	out_stroke_TIA

Acronyms: GDPPR (General Practice Extraction Service (GPES) Data for pandemic planning and research), HES (Hospital Episode Statistics), APC (Admitted Patient Care), , ADDE (Annual District Death Extract), PEDW (Patient Episode Dataset for Wales), WLGP (Welsh Longitudinal GP Dataset - Welsh Primary Care), PATD (COVID-19 Test Results), CHESS (COVID-19 Hospitalisation in England Surveillance System), SGSS (Covid-19 Second Generation Surveillance System).

UOB Open

Supplementary Table 3: Codelist for derivation of major outcomes and COVID-19 variables in the English NHS Digital TRE

All codelists are available in the GitHub for England https://github.com/BHFDSC/CCU002_01/tree/main/england/phenotypes and similarly for Wales where Read codes were used for primary care: https://github.com/BHFDSC/CCU002_01/tree/main/Wales/phenotypes

Outcome name	Codelist	Code type
**Arterial thromboses**		
**Acute myocardial infarction**	I21, I22, I23	ICD10
**Acute myocardial infarction**	1755008, 10273003, 15990001, 22298006, 42531007, 52035003, 54329005, 57054005, 58612006, 62695002, 65547006, 66189004, 70211005, 70422006, 73795002, 76593002, 79009004, 129574000, 161502000, 161503005, 194802003, 194809007, 194856005, 194857001, 194858006, 194862000, 194863005, 194865003, 194866002, 194867006, 194868001, 233838001, 233843008, 233846000, 233847009, 304914007, 307140009, 308065005, 311792005, 311793000, 311796008, 314116003, 315287002, 371068009, 398274000, 399211009, 401303003, 401314000, 428752002, 698593009, 15712881000119105, 15712921000119103, 233825009, 233826005, 233832000, 285991000119100, 394659003, 394710008, 428196007, 703213009, 1089471000000109, 12238151000119107, 233827001, 233828006, 233829003, 233830008, 233831007, 233833005, 233834004, 233835003, 314207007, 703164000, 703209002, 703211006, 703360004	SNOMED
**Ischaemic stroke**	G95.1, I63.1, I63.8, I63.3, I64, I63.4, I63.2, I63.9, I63.5, I63.0, H34	ICD10
**Ischaemic stroke**	71444005, 75543006, 78569004, 95457000, 95460007, 195185009, 195186005, 195189003, 195190007, 195200006, 195201005, 195209007, 195210002, 195211003, 230691006, 230692004, 230698000, 230699008, 230700009, 307766002, 307767006, 373606000, 413102000, 432504007, 724424009, 125081000119106, 140921000119102, 1089421000000105, 16000511000119103, 16002031000119102, 16002111000119106, 195230003, 20059004, 230693009, 230696001, 230701008, 307363008, 329641000119104, 371041009, 734383005, 87555007, 1089411000000104, 195213000, 230694003, 230695002, 230702001, 230703006, 230704000, 24654003, 25133001, 276219001, 276220007, 276221006, 276222004, 413758000, 426107000, 734384004, 90099008, 195212005, 195213000, 195216008, 195217004, 230690007, 275434003, 16371781000119100, 195209007, 195210002, 195211003, 230706003, 230708002, 281240008, 297138001, 444172003, 230707007, 230713003, 230714009, 230715005	SNOMED
**Other arterial embolism**	I74	ICD10
**Venous thromboembolic events**		
**Pulmonary embolism**	I26.9, I26.0	ICD10
**Lower limb deep vein thrombosis**	I80, O87.1, O87.9, O88.2, O22.3	ICD10
**Other deep vein thrombosis**	I82.3, I82.2, I82.9, I82.0, I82.8	ICD10

UOB Open

**Portal vein thrombosis**	I81	ICD10
**Intracranial venous thrombosis**	I67.6, G08, I63.6, O87.3, O22.5	ICD10
**Other vascular events**		
**Heart failure**	I13.2, I13.0, I11.0, I50	ICD10
**Heart failure**	717491000000102, 420300004, 430396006, 703272007, 446221000, 233924009, 10335000, 194767001, 83105008, 426611007, 275514001, 134440006, 314206003, 92506005, 43736008, 10633002, 88805009, 87837008, 195114002, 206586007, 56675007, 85232009, 426263006, 194781004, 194779001, 421704003, 407596008, 420913000, 422293003, 698592004, 134401001, 48447003, 367363000, 42343007, 79955004, 128404006, 195112003, 760361000000100, 195111005, 71892000, 84114007	SNOMED
**Angina**	I20	ICD10
**Angina**	15960581000119102, 10971000087107, 300995000, 414545008, 697976003, 413439005, 394659003, 371810009, 713405002, 315025001, 25106000, 314116003, 414795007, 225566008, 85284003, 233821000, 87343002, 89323001, 371807002, 61490001, 35928006, 429559004, 233819005, 59021001, 41334000, 19057007, 194828000, 371812001, 15960381000119109, 15960061000119102, 15960141000119102, 712866001, 791000119109, 371811008, 371809004, 703214003, 413838009, 194823009, 413844008, 233823002, 413444003, 46109009, 4557003, 371806006, 15960661000119107, 371808007, 21470009	SNOMED
**Subarachnoid haemorrhage and haemorrhagic stroke**	I60, I61	ICD10
**Confirmed COVID-19**	U07.1	ICD10
**Confirmed COVID-19**	1321211000000109, 1321341000000103, 1321801000000108, 1321811000000105, 1240511000000106, 1240571000000101, 1321241000000105, 1321551000000106, 1240581000000104, 1240401000000105, 1240391000000107, 1300681000000102, 1029481000000103, 1300631000000101, 1300671000000104, 121973000, 120814005, 1240381000000105, 1321191000000105, 1300721000000109, 1240541000000107, 1322781000000102, 1240751000000100, 1240561000000108, 1321541000000108, 1321181000000108, 1321321000000105, 1321761000000103, 1240411000000107, 1240741000000103, 1008541000000105, 186747009, 1321201000000107, 1300731000000106, 1321331000000107, 1321351000000100, 1321301000000101, 1321311000000104, 1322871000000109, 1240521000000100, 1240531000000103, 1240551000000105, 1240421000000101	SNOMED

UOB Open

Supplementary Table 4. Derivation of covariates in the English NHS Digital TRE and Welsh SAIL Databank TRE environments.

All codelists are available in the GitHub for England: github.com/BHFDSC/CCU002_01/tree/main/england/phenotypes
and similarly for Wales where Read codes were used for primary care: https://github.com/BHFDSC/CCU002_01/tree/main/Wales/phenotypes

Variable	Data Sources (NHS England)	Data Sources (SAIL Databank)	Description	Variable name in analysis code
**Demographic characteristics**				
Patient pseudoidentifier	All	All	Patient pseudoidentifier	NHS_NUMBER_DEID
death date	GDPPR, HES APC, deaths registry	C19_Cohort20 (WDSD, ADDE, ADDD, CDDS)	Earliest date of death recorded in specified data sources	death_date
sex	GDPPR, HES APC, HES OP, HES AE	C19_Cohort20 (WDSD)	Binary indicator for male or female (most recent record prior to index date and will initially select from primary care if possible)	cov_sex
age	GDPPR, HES APC, HES OP, HES AE	C19_Cohort20 (WDSD)	Age in years calculated on index date (will initially select from primary care if possible)	cov_age
ethnicity	GDPPR, HES APC, HES OP, HES AE	CCDS, CVVD, EDDS, ICNC, MIDS, NCCH, OPRD, PEDW, WASD	Patient ethnicity (most recent record prior to index date from primary care - Black or Black British, Asian or Asian British, Other, Mixed, Missing, Unknown)	cov_ethncity
smoking status	GDPPR	WLGP	Most recent smoking status (Smoker, Ex-smoker, Never-smoker) prior to index date	cov_smoking_status
deprivation	English indices of deprivation year 2019	C19_Cohort20 (WDSD, PEDW)	Most recent decile of deprivation prior to index date, defined using IMD	cov_deprivation
region of residence	English indices of deprivation year 2019	N/A	Most recent residence prior to index date (East of England, London, Midlands, NE and Yorkshire, North West, South East, South West, Scotland, Wales)	cov_region
**Medications**				
antiplatelet	primary care medications	WDDS	Binary indicator for antiplatelet medication with at least one prescription within 3 months prior to index date, defined using drug use	cov_antiplatelet_meds
lipid lowering	primary care medications	WDDS	Binary indicator for lipid lowering medication with at least one prescription within 3 months prior to index date, defined using drug use	cov_lipid_meds
anticoagulation	primary care medications	WDDS	Binary indicator for anticoagulation medication with at least one prescription within 3 months prior to index date, defined using drug use	cov_anticoagulation_meds
combined oral contraceptive pill	primary care medications	WDDS	Binary indicator for COCP medication with at least one prescription within 3 months prior to index date, defined using drug use	cov_cocp_meds
blood pressure lowering	primary care medications	WDDS	Binary indicator for bp lowering medication with at least one prescription within 3 months prior to index date, defined using drug use	cov_ever_icvt
hormone replacement therapy	primary care medications	WDDS	Binary indicator for HRT medication with at least one prescription within 3 months prior to index date, defined using drug use	cov_hrt_meds

UOB Open

Variable	Data Sources (NHS England)	Data Sources (SAIL Databank)	Description	Variable name in analysis code
**Past history of disease**				
acute myocardial infection	GDPPR, HES APC	WLGP, PEDW	Binary indicator for acute myocardial infarction prior to index date, defined using condition diagnosis code only	cov_ever_ami
embolism	GDPPR, HES APC	WLGP, PEDW	Binary indicator for pulmonary embolism or DVT prior to index date, defined using condition diagnosis code only	cov_ever_pe_vt
intracranial venous thrombosis	GDPPR, HES APC	WLGP, PEDW	Binary indicator for ICVT prior to index date, defined using condition diagnosis code only	cov_ever_icvt
any stroke	GDPPR, HES APC	WLGP, PEDW	Binary indicator for stroke (ischaemic stroke, intracranial haemorrhage, or stroke of unknown type) prior to index date, defined using condition diagnosis code only	cov_ever_all_stroke
thrombophilia	GDPPR, HES APC	WLGP, PEDW	Binary indicator for thrombophilia prior to index date, defined using condition diagnosis code only	cov_ever_thrombophilia
thrombocytopenia, TTP	GDPPR, HES APC	WLGP, PEDW	Binary indicator for thrombocytopenia prior to index date, defined using condition diagnosis code only	cov_ever_tcp
dementia	GDPPR, HES APC	WLGP, PEDW	Binary indicator for dementia prior to index date, defined using condition diagnosis code only	cov_ever_dementia
liver disease	GDPPR, HES APC	WLGP, PEDW	Binary indicator for liver disease prior to index date, defined using condition diagnosis code only	cov_ever_liver_disease
chronic kidney disease	GDPPR, HES APC	WLGP, PEDW	Binary indicator for chronic kidney disease prior to index date, defined using condition diagnosis code only	cov_ever_ckd
cancer	GDPPR, HES APC	WLGP, PEDW	Binary indicator for cancer prior to index date, defined using condition diagnosis code only	cov_ever_cancer
hypertension	GDPPR, HES APC, primary care medications	WLGP, PEDW, WDDS	Binary indicator for hypertension prior to index date, defined using either condition diagnosis code or drug use	cov_ever_hypertension
diabetes	GDPPR, HES APC, primary care medications	WLGP, PEDW, WDDS	Binary indicator for diabetes prior to index date, defined using either condition diagnosis code or drug use	cov_ever_diabetes
obesity	GDPPR, HES APC	WLGP, PEDW	Binary indicator for obesity prior to index date, defined using either condition diagnosis code code or measurement	cov_ever_obesity
depression	GDPPR, HES APC	WLGP, PEDW	Binary indicator for depression prior to index date, defined using condition diagnosis code only	cov_ever_depression
COPD	GDPPR, HES APC	WLGP, PEDW	Binary indicator for chronic obstructive pulmonary disease prior to index date, defined using condition diagnosis code only	cov_ever_copd
number of unique disorders	GDPPR	WLGP	Number of consultations for different ailments/conditions/diseases in the year prior to index date	cov_n_disorder

UOB Open

Variable	Data Sources (NHS England)	Data Sources (SAIL Databank)	Description	Variable name in analysis code
disseminated intravascular coagulation	GDPPR, HES APC	WLGP, PEDW	Binary indicator for disseminated intravascular coagulation prior to index date, defined using condition diagnosis code only	cov_ever_other_arterial_embolism
mesenteric thrombus	GDPPR, HES APC	WLGP, PEDW	Binary indicator for mesenteric thrombus prior to index date, defined using condition diagnosis code only	cov_ever_dic
artery dissection	GDPPR, HES APC	WLGP, PEDW	Binary indicator for artery dissection prior to index date, defined using condition diagnosis code only	cov_ever_mesenteric_thrombus
life threatening arrhythmias	GDPPR, HES APC	WLGP, PEDW	Binary indicator for life threatening arrhythmias prior to index date, defined using condition diagnosis code only	cov_ever_artery_dissect
cardiomyopathy	GDPPR, HES APC	WLGP, PEDW	Binary indicator for cardiomyopathy prior to index date, defined using condition diagnosis code only	cov_ever_life_arrhythmia
heart failure	GDPPR, HES APC	WLGP, PEDW	Binary indicator for heart failure prior to index date, defined using condition diagnosis code only	cov_ever_cardiomyopathy
pericarditis	GDPPR, HES APC	WLGP, PEDW	Binary indicator for pericarditis prior to index date, defined using condition diagnosis code only	cov_ever_hf
myocarditis	GDPPR, HES APC	WLGP, PEDW	Binary indicator for myocarditis prior to index date, defined using condition diagnosis code only	cov_ever_pericarditis
major surgery in the last year	HES APC	PEDW	Binary indicator for major surgery within a year prior to index date, defined using condition diagnosis code only	cov_ever_myocarditis

Acronyms: GDPPR (General Practice Extraction Service (GPES) Data for Pandemic Planning and Research), HES (Hospital Episode Statistics), APC (Admitted Patient Care), AE (Accident and Emergency), WDSD (Welsh Demographic Service Dataset), ADDE (Annual District Death Extract), ADDD (Annual District Death Daily), CDDS (COVID-19 Consolidated Deaths), CCDS (Critical Care Dataset), CVVD (Covid Vaccination Dataset), EDDS (Emergency Department Dataset), ICNC (Intensive Care National Audit and Research Centre - Covid19), MIDS (Maternity Indicators Dataset), NCCH (National Community Child Health), OPRD (Outpatient Referral), PEDW (Patient Episode Dataset for Wales), WASD (Welsh Ambulance Services NHS Trust), WLGP (Welsh Longitudinal GP Dataset - Welsh Primary Care), WDDS (Welsh Dispensing Dataset).

UOB Open

Supplementary Table 5. Number of patients analysed in the Welsh SAIL Databank and the number of people (risk per 100,000 during follow up) who were and were not hospitalised within 28 days of diagnosis of COVID-19.

Characteristic		All	Diagnosed with COVID-19 Hospitalised N (per 100,000)	Diagnosed with COVID-19 Non-hospitalised N (per 100,000)
**All**		2,615,854	7,106 (272)	71,606 (2,737)
**Sex**	Male	1,297,835	3,778 (292)	30,312 (2,336)
	Female	1,318,019	3,328 (253)	41,294 (3,133)
**Age in decades**	18-29	480,469	128 (26.6)	18,090 (3765)
	30-39	416,949	241 (57.8)	13,063 (3133)
	40-49	395,769	522 (132)	12,904 (3261)
	50-59	458,144	880 (192)	13,312 (2906)
	60-69	379,682	1,095 (288)	6,081 (1602)
	70-79	309,639	1,737 (561)	3,690 (1192)
	80-89	144,208	1,881 (1304)	3,163 (2193)
	90+	30,994	622 (2007)	1,303 (4204)
**Ethnicity**	Asian or Asian British	42,731	123 (288)	1,689 (3953)
	Black or Black British	11,921	22 (185)	414 (3473)
	Missing	489,772	593 (121)	11,479 (2344)
	Mixed	76,114	323 (424)	2,475 (3252)
	Other Ethnic Groups	15,174	36 (237)	435 (2867)
	White	1,980,142	6,009 (304)	55,114 (2783)
**Index of Multiple Deprivation**	1 (most deprived)	494,007	1,120 (227)	12,459 (2522)
	2	489,758	1,098 (224)	10,886 (2223)
	3	492,736	1,158 (235)	11,788 (2392)
	4	485,216	1,562 (322)	15,680 (3232)
	5 (least deprived)	478,180	1,758 (368)	15,999 (3346)
	Missing	175,957	410 (233)	4,794 (2725)
**Smoking status**	Current	560,828	1,087 (194)	12,399 (2211)
	Former	160,367	903 (563)	3,869 (2413)
	Missing	512,977	1,121 (219)	10,795 (2104)
	Never	1,381,682	3,995 (289)	44,543 (3224)
**Medical history**	Arterial thrombosis	120,085	1,536 (1,279)	2,937 (2446)
	Venous thromboembolisum	38,072	449 (1179)	1,033 (2713)
**Number of medications**	0	1,079,900	730 (67.6)	28,924 (2678)
	1-5	1,367,052	4,060 (297)	37,944 (2,776)
	6+	168,902	2,316 (1371)	4,738 (2805)
**Number of diagnoses**	0	2,219,689	3,969 (179)	61,083 (2,752)
	1-5	248,262	2,091 (842)	6,974 (2,809)
	6+	147,903	1,046 (707)	3,549 (2,400)

UOB Open

Supplementary Table 6. Hazard ratios (95% CI) compared with no COVID-19 for first arterial thrombosis and first venous thromboembolism, according to time since diagnosis of COVID-19. All results are maximally adjusted.

	Weeks since COVID-19 diagnosis						
	**1**	**2**	**3-4**	**5-8**	**9-12**	**13-26**	**27-49**
Arterial thrombosis							
Critical care within 28 days of diagnosis	-	-	-	9.09 (7.19-11.5)	4.91 (3.43-7.04)	2.30 (1.70-3.09)	1.79 (1.14-2.81)
No critical care within 28 days of diagnosis	-	-	-	2.71 (2.37-3.09)	1.71 (1.42-2.07)	1.44 (1.29-1.62)	1.43 (1.22-1.67)
Fatal event	18.6 (17.3-20.2)	6.26 (5.43-7.22)	3.95 (3.44-4.54)	2.32 (2.00-2.70)	1.87 (1.54-2.26)	1.82 (1.62-2.05)	1.40 (1.13-1.75)
Non-fatal event	20.0 (19.2-20.7)	3.11 (2.81-3.43)	2.44 (2.24-2.66)	1.85 (1.71-2.00)	1.50 (1.35-1.66)	1.19 (1.11-1.28)	1.29 (1.15-1.45)
Venous thromboembolism							
Critical care within 28 days of diagnosis	-	-	-	31.4 (24.2-40.8)	11.0 (6.73-18.0)	3.52 (2.18-5.67)	1.10 (0.36-3.42)
No critical care within 28 days of diagnosis	-	-	-	8.78 (7.41-10.4)	4.54 (3.51-5.89)	2.56 (2.11-3.11)	1.54 (1.09-2.17)
Fatal event	21.1 (17.3-25.7)	6.64 (4.60-9.58)	5.00 (3.61-6.93)	2.68 (1.85-3.87)	2.42 (1.54-3.82)	2.05 (1.52-2.77)	0.72 (0.32-1.61)
Non-fatal event	22.5 (20.9-24.2)	6.78 (5.90-7.78)	7.78 (7.07-8.56)	4.39 (3.95-4.87)	2.91 (2.50-3.40)	2.13 (1.91-2.38)	1.82 (1.48-2.23)

UOB Open

Supplementary Figure 1. Maximally adjusted hazard ratios (log scale) for different arterial thrombotic, and venous thromboembolic and other vascular events by time since diagnosis of COVID-19, separately for hospitalised and non-hospitalised COVID-19.

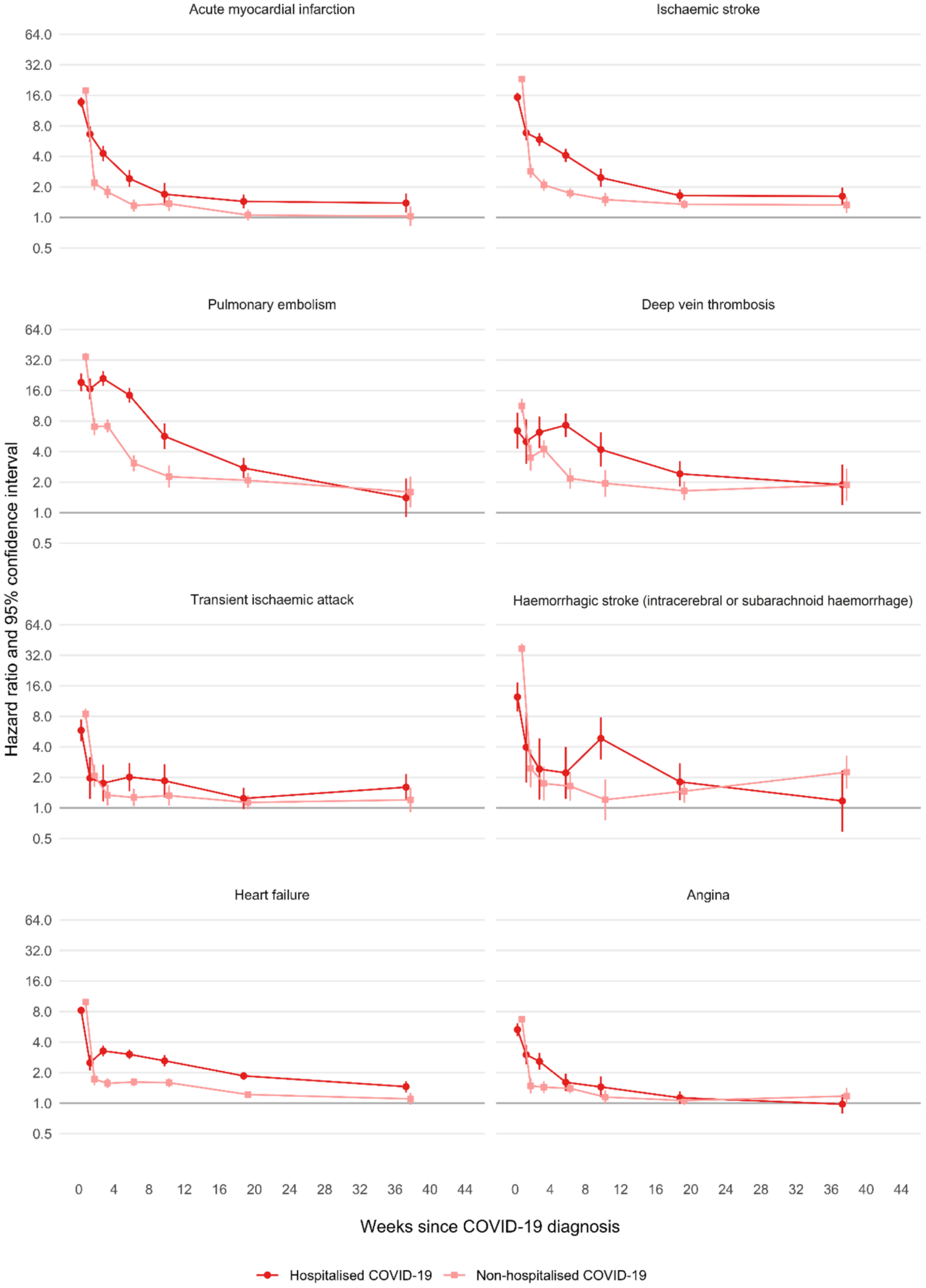

UOB Open

Supplementary Figure 2. Maximally adjusted hazard ratios (log scale) for first arterial thrombosis and first venous thromboembolism by time since diagnosis of COVID-19, separately for those who received critical care for COVID-19 within 28 days of diagnosis and those who did not receive critical care for COVID-19 within 28 days of diagnosis.

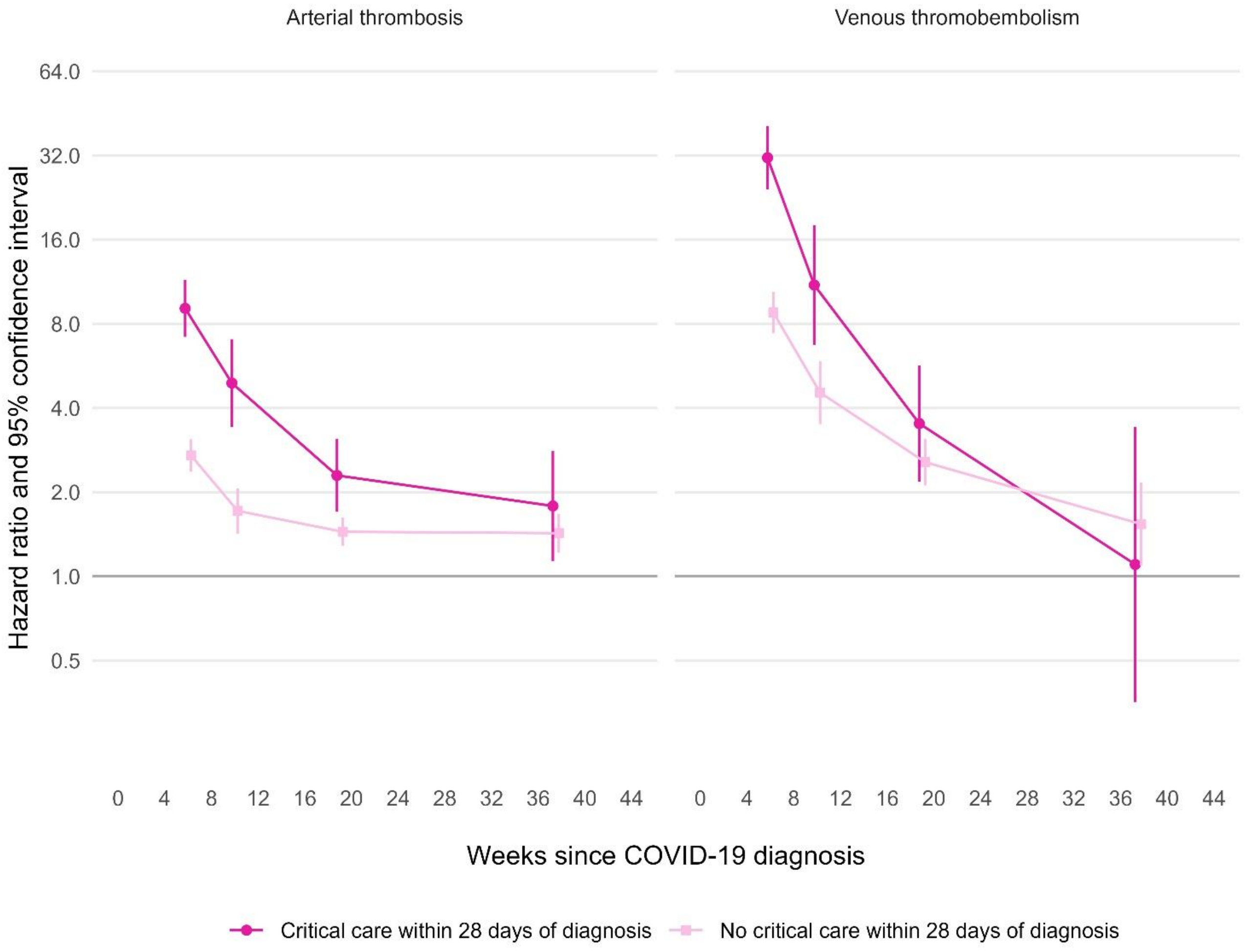

UOB Open

Supplementary Figure 3. Maximally adjusted hazard ratios (log scale) for first arterial thrombosis and first venous thromboembolism different by time since diagnosis of COVID-19, separately for fatal and non-fatal events.

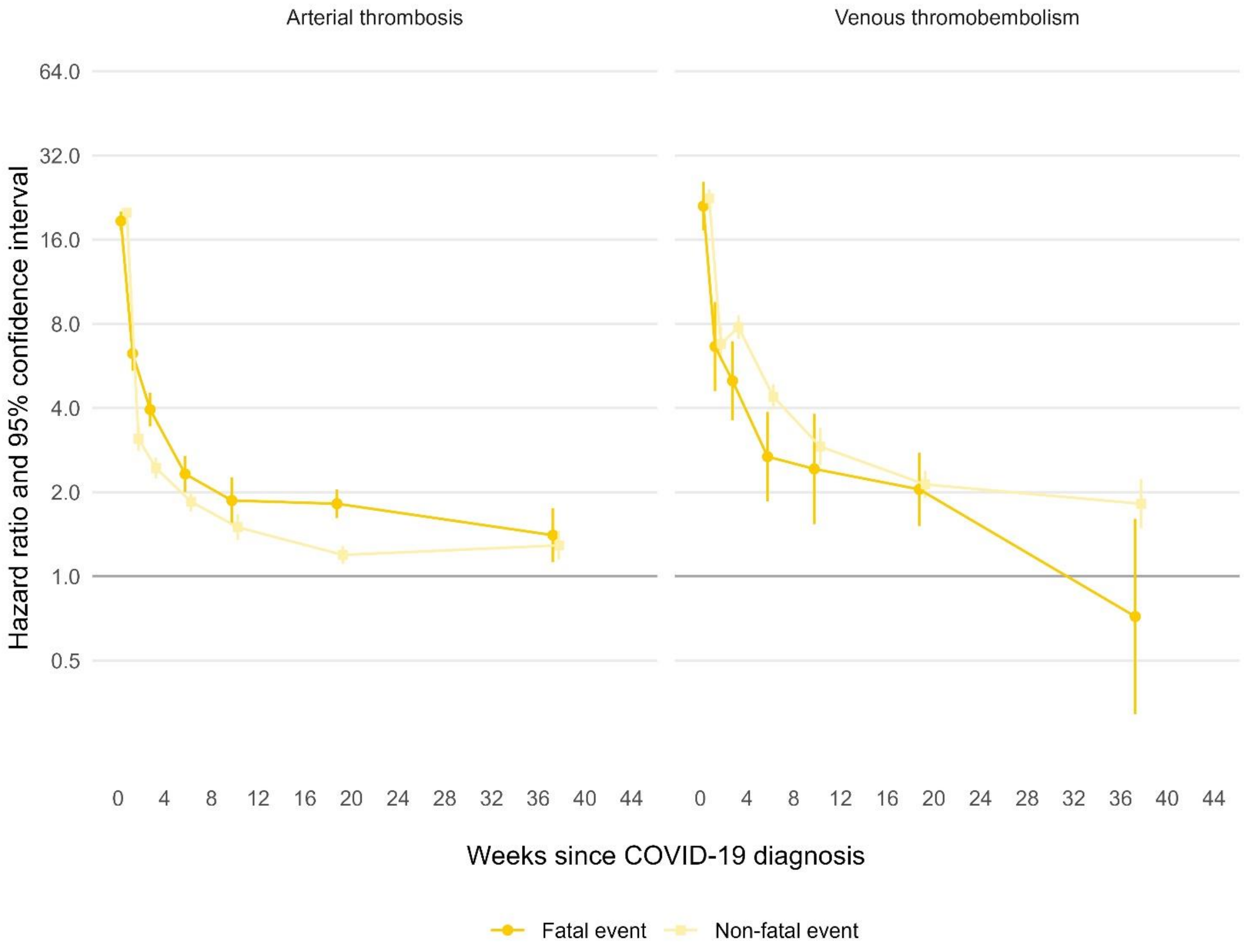

UOB Open

Supplementary Figure 4. Estimated absolute increase in risk of arterial thromboses and venous thromboembolisms over time since diagnosis of COVID-19, compared with no COVID-19 diagnosis, after excluding the risk during the first 28 days since diagnosis

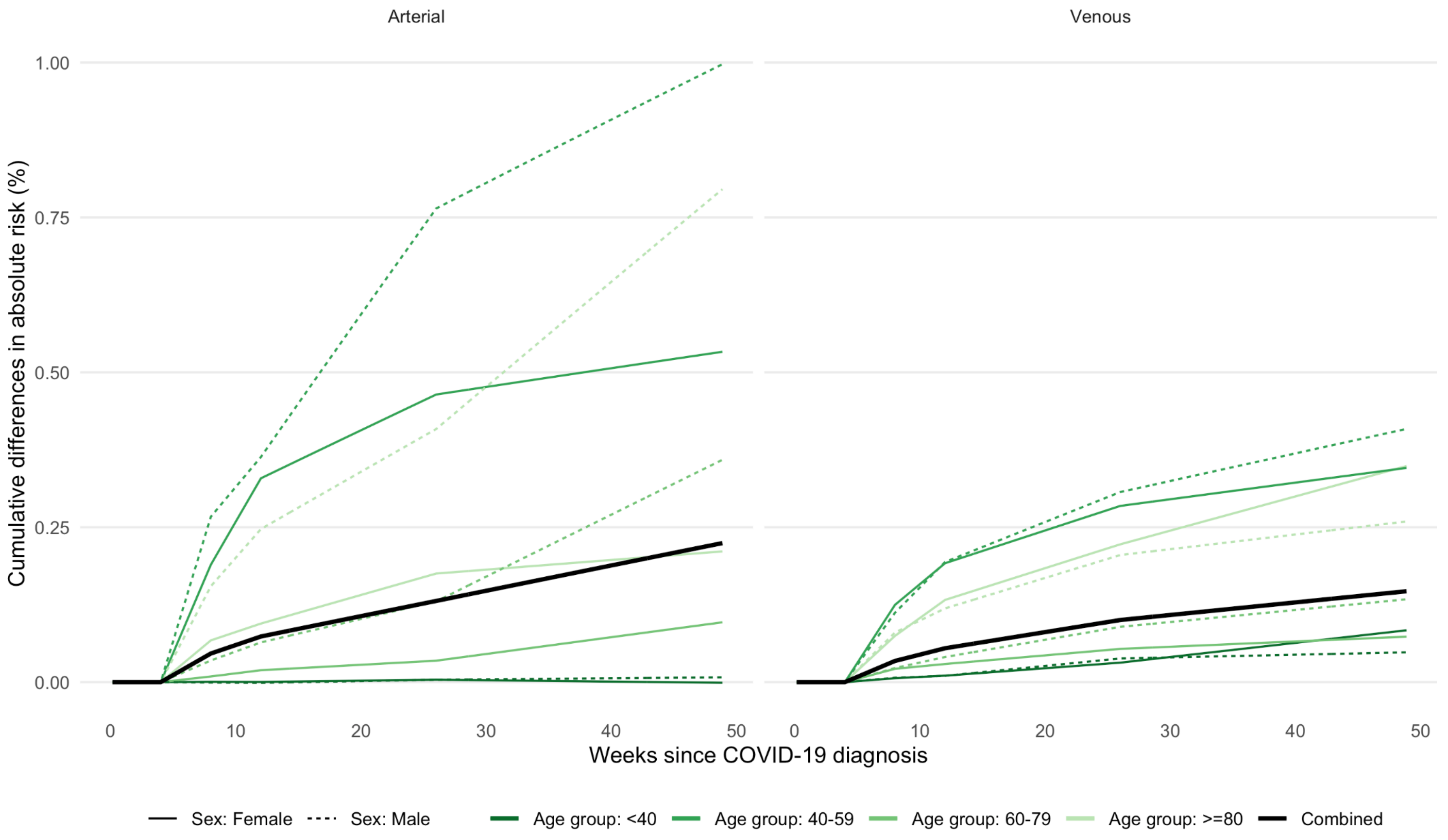

UOB Open

Supplementary Figure 5. Estimated absolute increase in risk of arterial thromboses and venous thromboembolisms over time since diagnosis of COVID-19, compared with no COVID-19 diagnosis, stratified by those with hospitalised COVID-19 and non-hospitalised COVID-19.

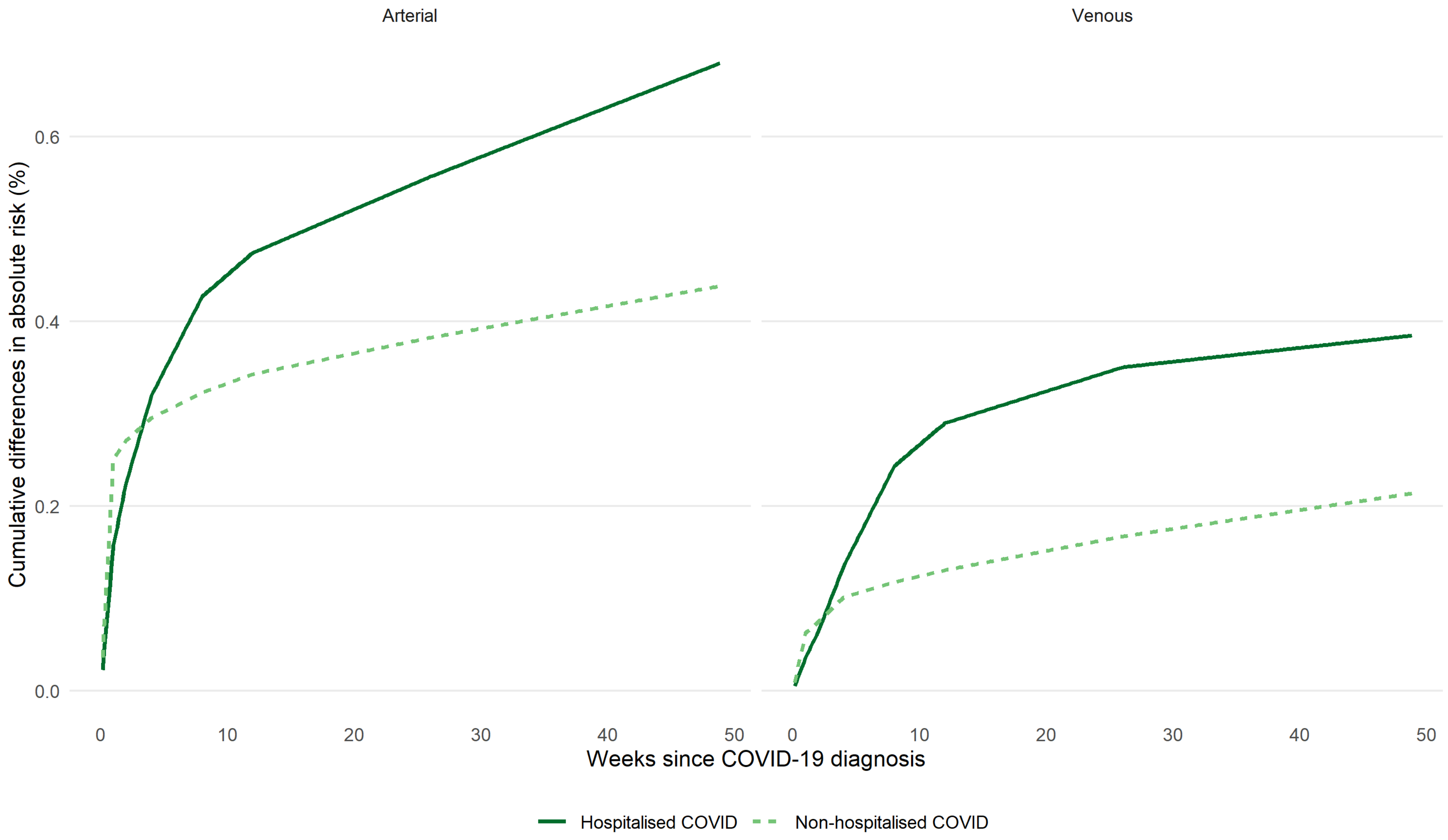

UOB Open
